# Selection of Single Domain Antibodies from Immune Libraries Displayed on the Surface of *E. coli* Cells with Two β-Domains of Opposite Topologies

**DOI:** 10.1371/journal.pone.0075126

**Published:** 2013-09-23

**Authors:** Valencio Salema, Elvira Marín, Rocio Martínez-Arteaga, David Ruano-Gallego, Sofía Fraile, Yago Margolles, Xema Teira, Carlos Gutierrez, Gustavo Bodelón, Luis Ángel Fernández

**Affiliations:** 1 Department of Microbial Biotechnology, Centro Nacional de Biotecnología, Consejo Superior de Investigaciones Científicas (CSIC), Campus UAM Cantoblanco, Madrid, Spain; 2 Department of Animal Medicine and Surgery, Veterinary Faculty, Universidad de Las Palmas de Gran Canaria (UPGC), Las Palmas, Canary Islands, Spain; Louisiana State University, United States of America

## Abstract

Screening of antibody (Ab) libraries by direct display on the surface of *E. coli* cells is hampered by the presence of the outer membrane (OM). In this work we demonstrate that the native β-domains of EhaA autotransporter and intimin, two proteins from enterohemorrhagic *E. coli* O157:H7 (EHEC) with opposite topologies in the OM, are effective systems for the display of immune libraries of single domain Abs (sdAbs) from camelids (nanobodies or V_HH_) on the surface of *E. coli* K-12 cells and for the selection of high affinity sdAbs using magnetic cell sorting (MACS). We analyzed the capacity of EhaA and intimin β-domains to display individual sdAbs and sdAb libraries obtained after immunization with the extracellular domain of the translocated intimin receptor from EHEC (TirM_EHEC_). We demonstrated that both systems displayed functional sdAbs on the surface of *E. coli* cells with little proteolysis and cellular toxicity, although *E. coli* cells displaying sdAbs with the β-domain of intimin showed higher antigen-binding capacity. Both *E. coli* display libraries were screened for TirM_EHEC_ binding clones by MACS. High affinity binders were selected by both display systems, although more efficiently with the intimin β-domain. The specificity of the selected clones against TirM_EHEC_ was demonstrated by flow cytometry of *E. coli* cells, along with ELISA and surface plasmon resonance with purified sdAbs. Finally, we employed the *E. coli* cell display systems to provide an estimation of the affinity of the selected sdAb by flow cytometry analysis under equilibrium conditions.

## Introduction

The expression of antibodies (Abs) in *E. coli*, both full-length immunoglobulin G (IgG) molecules and smaller antigen-binding fragments containing the variable (V) domains from heavy (H) and/or light (L) chains e.g. Fab, single-chain Fv (scFv), and single domain Ab (sdAb), provides a set of powerful technologies for the generation of Abs with novel specificities and improved properties [1,2]. Current selection of novel therapeutic Abs is based on hybridoma technologies using transgenic mice carrying human Ig genes [3] and screening of Ab gene libraries displayed on the surface of a biological entity [4].

The most common Ab display method is phage display, in which the V-genes are cloned in phagemids as fusions to the minor coat protein III (pIII) from filamentous bacteriophages of *E. coli* [5]. The Ab-pIII fusions contain a N-terminal signal peptide (SP) to translocate the Ab to the periplasm while the pIII moiety is anchored in the inner membrane (IM) [6]. Abs expressed in the periplasm of *E. coli* generally fold properly due to the presence of protein chaperones (e.g. Skp, FkpA) and disulfide bond forming and isomerization enzymes (e.g. DsbA, DsbC) [7]. Further, infection of *E. coli* cells expressing Ab-pIII fusions with a helper bacteriophage allows the production of phage particles displaying the Ab (Phabs), which can be incubated with the antigen of interest to recover antigen binding clones and amplified by infection of fresh *E. coli* cells (a process called biopanning).

An alternative technology for Ab display and selection in *E. coli* is the anchored periplasmic expression (APEx), in which the Ab fragments or full-length IgGs are expressed in the periplasm and are tethered to the IM by means of a short lipoprotein signal or an engineered lipoprotein binding the Fc region of IgGs [8,9]. In APEx, the outer membrane (OM) of *E. coli* is permeabilized and the generated spheroplasts are incubated with the antigen labeled with a fluorophore or biotin, and subsequently selected by fluorescence activated cell sorting (FACS).

Although phage display and APEx are robust technologies for Ab selection, alternative methods that enable the direct display of Abs or Ab libraries on the surface of *E. coli* cells, without the need for generation of Phabs or spheroplasts would be of great interest. In addition, *E. coli* cell display would facilitate selections by cell sorting methods using antigen in solution as well as the analysis of the selected clones by flow cytometry. Alternative successful cell display technologies developed for Ab selection utilize yeasts [10,11] and Gram-positive bacteria [12]. In these cell display systems, the Ab fragments translocate across a single cell membrane and are anchored in the cell wall. Nevertheless, *E. coli* remains a more suitable microorganism for the generation, amplification and maintenance of large Ab repertoires owing to its high-efficiency of transformation and versatile expression systems. Despite these advantages, the presence of the OM has hindered the development of effective *E. coli* cell display methods for Ab selection, with the exception of the use of the chimeric lipoprotein Lpp-OmpA' for the display of scFvs and selection of variants with higher affinity after mutagenesis of the scFv (affinity maturation) [13-15]. Lpp-OmpA' consists of the N-terminal SP and first 9 residues of the mature Lpp fused to residues 46 to 159 of OmpA, which is a truncated fragment of its native 8-stranded β-barrel [16]. However, this chimeric construct lacks the characteristic stability of the β-barrel of native OM proteins (OMPs) [17] and its expression induces OM leakage as well as cellular toxicity [18,19], which may have limited its use to the affinity maturation of scFvs.

Other OMPs have also been used to display heterologous peptides and proteins on the surface of *E. coli* cells [20]. Among them, the autotransporters (AT) and Intimin/Invasin (Int/Inv) proteins are very attractive display systems [21,22]. Interestingly, an AT protein (EspP) has been used in *E. coli* for the display and affinity maturation of an Anticalin protein scaffold binding human cytotoxic T-lymphocyte antigen 4 (CTLA-4) [23]. Protein members of the AT and Int/Inv families are large, secreted polypeptides that contain three functional regions: i) a N-terminal SP, that drives their Sec-dependent translocation across the IM; ii) a β-domain, that is anchored into the OM and comprises a 12-stranded β-barrel with a peptide linker running through the lumen of the β-barrel; and iii) a *passenger* region, that is secreted to the extracellular milieu [24]. Although their mechanism of secretion remains uncertain, AT and Int/Inv proteins are translocated into the periplasm and then use the β-barrel assembly machine (BAM) complex for insertion into the OM and translocation of the *passenger* region to the cell surface [24-26].

**Figure 1:**
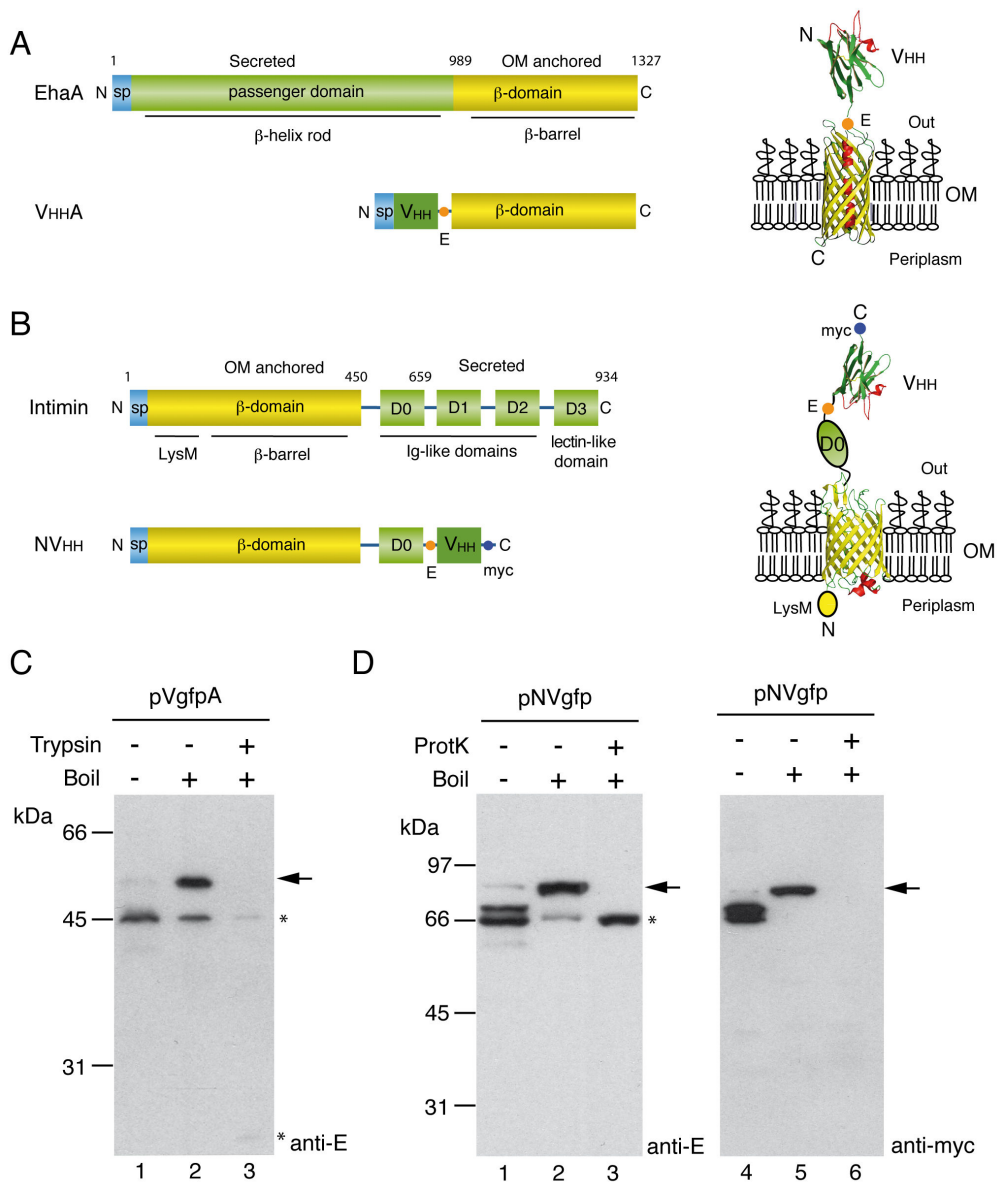
*E. coli* cell surface display of VHH sdAbs with EhaA and Intimin β-domains. (**A**) Scheme of EhaA autotransporter and VHHA fusions (left), showing N-terminal SP, secreted passenger or VHH domain, and C-terminal β-domain. Model of VHHA fusion in the OM (right), with N-terminal VHH domain exposed to the extracellular milieu and with C-EhaA β-barrel inserted in the OM. These domains are connected with the E-tag epitope and the internal α-helical linker of the β-barrel. (**B**) Scheme of Intimin and NVHH fusions (left), showing N-terminal SP, LysM and β-domains, and secreted D0-D3 Ig-like and lectin-like domains, or VHH domain replacing D1-D3 in NVHH fusions. Model of NVHH fusion in the OM (right), with N-terminal LysM domain in the periplasm, β-barrel with linker in the OM, and connecting with C-terminal D0 and VHH domains exposed to the extracellular milieu. The E-tag and myc-tag epitopes flanking the VHH domain are indicated. (**C**) and (**D**) Western blots of whole-cell protein extracts from induced *E. coli* UT5600 harbouring pVgfpA (C) or pNVgfp (D). Intact *E. coli* cells were incubated with (+) or without (-) the indicated protease, Trypsin or Proteinase-K (ProtK), before lysis. Protein extracts were prepared in SDS (C) or SDS-urea (D) sample buffers and boiled (+) or not boiled (-) before SDS-PAGE. Western blots were developed with anti-E or anti-myc mAb, as indicated. The positions of full-length VgfpA and NVgfp fusions are labeled with arrows. Asterisks indicate protein bands detected in protease-treated samples. The mass of protein markers (in kDa) is shown on the left.

Despite their similarities, AT and Int/Inv proteins also have important differences. Firstly, they have opposite topological organization in the OM, being the *passenger* region located in the N-terminal portion of ATs whereas in the case of Int/Inv proteins is found in the C-terminus (Figure 1). The distinct topologies are also reflected in their β-domains. In the case of ATs, the β-barrel is preceded by α-helix linker that fills the lumen and connects its N-terminus with the *passenger* region [27,28]. In contrast, the β-barrel of Int/Inv proteins is followed by a peptide linker that runs through the lumen connecting its C-terminus to the *passenger* region [29]. In addition, the *passengers* of AT and Int/Inv proteins also have distinct structures, namely β-helical rods in most ATs and tandems of Ig-like domains in Int/Inv proteins [30,31].

In previous works, we have studied the secretion mechanism of ATs and reported their capacity to translocate a model sdAb to the surface of *E. coli* fused to the β-domain of ATs such as the IgA protease (IgAP) from *Neisseria gonorrhoeae* and EhaA from enterohemorrhagic *E. coli* (EHEC) O157:H7 [32,33]. A model sdAb fused to these β-domains was correctly folded in the periplasm with the canonical disulfide bond of Ig domains formed by the action of DsbA [32,33]. In a different study, we demonstrated that the Intimins from EHEC and enteropathogenic *E. coli* (EPEC) could be expressed in *E. coli* K-12 and displayed their native *passenger* domains on the cell surface with a disulfide bond formed by DsbA [25]. However, neither the display of heterologous Ig domains (e.g. sdAbs) fused to Intimin, nor the utility of the β-domains of ATs and Intimin for display of sdAb libraries and *de novo* selection of sdAbs against an antigen of interest was investigated.

In this work, we have demonstrated the capacity of the β-domains of EhaA and Intimin for the display of sdAb libraries on the surface of *E. coli* cells and for the selection of novel sdAbs against the extracellular domain of the translocated intimin receptor from EHEC (TirM_EHEC_). *E. coli* display libraries were screened by magnetic cell sorting (MACS) and clones that specifically bound TirM_EHEC_ were isolated, characterized by flow cytometry, as well as by enzyme linked immunosorbent assay (ELISA) and surface plasmon resonance (SPR) with the purified sdAb. In addition, *E. coli* display was used to provide an estimation of the affinity of the selected sdAb by flow cytometry analysis under equilibrium conditions before protein purification.

## Materials and Methods

### Bacterial strains, bacteriophages, growth and induction conditions

The *E. coli* strains used in this work are listed in Table 1. Bacteria carrying plasmids with VHH were grown at 30 °C in Luria-Bertani (LB) liquid medium or on agar plates with the appropriate antibiotic for plasmid selection. LB plates and pre-inoculum media prior to induction contained 2% (w/v) glucose for repression of the *lac* promoter. The preinocula cultures were started from individual colonies (for single clones) or from a mixture of clones (in case of libraries), freshly grown and harvested from plates, diluted to an initial OD_600_ of 0.5, and grown overnight (o/n) under static conditions. For induction, bacteria (corresponding to an OD_600_ of 0.5) were harvested by centrifugation (4000 xg, 5 min), and grown in the same media with 0.05 mM isopropylthio-β-D-galactoside (IPTG), but without glucose for 3 h with agitation (160 rpm), unless indicated otherwise. For over-expression of soluble VHH in the periplasm, *E. coli* WK6 cells with the corresponding pCANTAB6-VHH plasmid (Ap^R^) were induced with 0.3 mM IPTG for 3 h at 30 °C. Secretion of VHH into the culture media was performed using the hemolysin (Hly) secretion system of *E. coli* HB2151 cells carrying pVDL9.3 (HlyBD; Cm^R^) and the corresponding pEHlyA4SD-VHH (Ap^R^) plasmids, induced with 0.3 mM IPTG for 6 h [34,35]. Over-expression of soluble TirM_EHEC_ with N-terminal His-tag was induced in *E. coli* BL21 (DE3) cells carrying the pET28a-TirM_EHEC_ plasmid (Km^R^) and grown at 37 °C in LB medium containing 1.0 mM IPTG for 2 h. Further details can be found in Materials and Methods S1.

**Table 1:** *E. coli* strains and plasmids.

**Name**	**Genotype and relevant properties**	**Reference**
***E. coli* strains**		
BL21 (DE3)	F^-^ *omp*T *hsd*S_B_(rB-, mB-) *gal dcm lon* λ(DE3 [*lacI* lacUV5-T7 *gene1 ind1 sam7 nin5*])	Novagen
DH10B-T1^R^	F^-^ *mcrA* Δ*mrr-hsdRMS-mcrBC φ80lacZDM15 ΔlacX74 recA1 endA1 araD139* Δ(*ara, leu*)*7697 galU galK rpsL* (*StrR*) *nupG tonA* λ^-^	Invitrogen
MG1655	K-12 (F^-^ λ^-^)	[61]
EcM1	MG1655Δ*fim*A-H	[62]
UT5600	K-12 (F^-^ λ^-^) Δ(*ompT-fepC*)*266*	[63]
WK6	Δ(*lac-proAB*), *galE*, *strA*, *nal*, F’[*lacI* ^q^ *Z*ΔM15, *proAB*]	[64]
HB2151	Δ(*lac-proAB*), *ara*, *nal* ^*R*^, *thi*, F’[*lacI* ^q^ *Z*ΔM15, *proAB*]	[65]
**Plasmids**		
pAK-Not	(Cm^R^), lacI^q^-P*lac* promoter, pBR322 ori	[66]
pHEA	pAK-Not derivative; for fusions to C-EhaA (pelB-His-E-tag-EhaA989-1327)	[33]
pVgfpA	pHEA-derivative; Vgfp fused to C-EhaA (pelB-Vgfp-E-tag-EhaA989-1327)	This work
pVTIRnA	pHEA-derivative; VTIR (n clone) fused to C-EhaA (pelB-VTIRn-E-tag-EhaA989-1327)	This work
pNeae	pAK-Not derivative; Neae[Intimin_EHEC_ (1-659)-E-His-tag]	[25]
pNeae2	pNeae-derivative; for fusions to Neae-myc [Intimin_EHEC_ (1-659)-E-His-myc tag]	This work
pNVgfp	pNeae-myc-derivative; NVgfp fusion [Intimin_EHEC_ (1-659)-E-Vgfp-myc tag]	This work
pNVTIRn	pNeae-myc-derivative; NVTIR (n clone) fusion [Intimin_EHEC_ (1-659)-E-VTIRn-myc tag]	This work
pCANTAB6	(Ap^R^), pUC-ori, for pIII fusions or soluble expression of sdAb with His-myc-tags	[67]
pCANTAB6-VTIR1	pCANTAB6-derivative; for expression of sdAb VTIR1 with His-myc-tags	This work
pCANTAB6-Vgfp	pCANTAB6-derivative; for expression of sdAb Vgfp with His-myc-tags	This work
pET28-a	(Km^R^), pBR322-ori, T7 promoter; for N-terminal His-tag protein fusions	Novagen
pET28-a-TirM_EHEC_	pET28-a derivative; His-tagged TirM_EHEC_ (residues 252 to 360 of Tir_EHEC_)	This work
pVDL9.3	(Cm^R^), pSC101-ori, *lac* promoter, for production of HlyB and HlyD transporters	[34]
pEHlyA2SD	(Ap^R^), pUC-ori, *lac* promoter, C-terminal E-tagged HlyA signal	[34]
pEHlyA4SD	pEHlyA2SD derivative with modified polylinker having unique *Sfi*I and *Not*I sites	This work
pEHlyA4SD-VTIRn	pEHlyA4SD derivative; VTIR (n clone) fused to C-terminal E-tagged HlyA signal	This work

### Plasmids, DNA constructs and oligonucleotides

Plasmids used in this study are summarized in Table 1. DNA manipulation, ligation, transformation and plasmid preparation were performed following standard techniques. Oligonucleotides were synthesized by Sigma Genosys, except those used for VHH amplification, which were from Scandinavian Gene Synthesis (SGS). All DNA constructs were sequenced by Secugen SL. PCR reactions for cloning were performed with proof-reading Vent DNA polymerase (New England Biolabs) or Taq DNA polymerase (Roche). The plasmid pHEA (Cm^R^), for in frame fusions to N-terminal PelB signal peptide and E-tagged C-EhaA (amino acids 989-1327 of EhaA from EHEC O157:H7 strain EDL933*stx*-), was reported previously [33]. The amino acid sequence of the E-tag is: GAPVPYPDLEPA. The plasmid pNeae2 (Cm^R^) is a derivative of the pNeae vector [25], encoding Intimin residues 1-659 (from EHEC O157:H7 strain EDL933*stx*-) followed by the E-tag, the hexahistidine (His) epitope, and a C-terminal myc-tag (EQKLISEED). The details of plasmid constructions are described in Materials and Methods S1.

### Immunization and generation of VHH sdAb libraries

Dromedary camel immunization ( *Camelus* *dromedarious* ) was performed with purified TirM_EHEC_ (~1 mgr) mixed with veterinary vaccine adjuvant (GERBU). Amplification of V_HH_ genes was done by RT-PCR of the mRNA isolated from ~2x10^7^ lymphocytes from a peripheral blood sample of the immunized animal using standard methods and primers [36]. The camel immunization protocol followed the animal experimentation guidelines published by the Canary Islands Regional Government (Spain) and was approved by the Ethics Commission of the Department of Animal Medicine and Surgery, University of "Las Palmas de Gran Canaria" (Spain). The amplified VHH fragments were digested with *Sfi*I and *Not*I restriction enzymes and ligated into the same sites of purified pHEA and pNeae2 backbone vectors and finally transformed in *E. coli* EcM1 cells by electroporation. The size of each library was ~2-3x10^6^ clones, as determined by plating on LB-Cm agar plates with 2% w/v glucose incubated at 30 °C. Further details are provided in t Materials and Methods S1.

### Protein extracts, SDS-PAGE and Western blots

Whole cell protein extracts were prepared by harvesting bacteria after induction (1 ml of OD_600_ 1.5), resuspended in 50 µl of 10 mM Tris HCl pH 8.0, mixed with the same volume of SDS-sample buffer (2X) or urea-SDS sample buffer (2X) and boiled for 10 min (pHEA constructs) or 30 min (pNeae constructs) respectively. The boiled samples were sonicated (5 sec; Labsonic B Braun), centrifuged (14,000 x*g*, 5 min) to pellet insoluble material, loaded onto 8% or 10% SDS-PAGE gels and run using a Miniprotean III electrophoresis system (Bio-Rad). For Western blot, the gels were transferred to a polyvinylidene difluoride membrane (PVDF, Immobilon-P, Millipore) using a semi-dry electrophoresis transfer apparatus (Bio-Rad), the membranes were blocked and incubated with anti-E-tag mAb (Phadia) or anti-c-myc-POD mAb (clone 9E10; Roche), as indicated. Bound anti-E-tag mAb was developed using anti-mouse IgG conjugated with peroxidase (POD) (Sigma). Streptavidin-POD conjugate (Roche) was employed to detect the biotinylated broad range SDS-PAGE protein markers (Bio-Rad). All mAbs and POD conjugates were used in a 1:5000 dilution. Membranes were developed by chemiluminescence and either exposed to an X-ray film (Curix, Agfa) or scanned in a Chemi-Doc XRS (Bio-Rad) and analyzed using the Quantity One software (Bio-Rad). To quantify the total number of V_HH_ fusions expressed in *E. coli*, Western blots of whole cell protein extracts and dilutions of a purified E-tagged V_HH_ of known concentration [37] (hereafter referred to as “unknowns” and “standard”, respectively) were visualized on a ChemiDoc XRS and analyzed using the Quantity One software (Bio-Rad). Composition of buffers and further details are described in Materials and Methods S1.

### Protease accessibility assays

Induced bacteria (1 ml, OD_600_=1.5) were harvested by centrifugation (4000 xg, 3 min) and resuspended in 100 µl of 10 mM Tris HCl pH 8.0. This bacterial suspension was incubated with trypsin (10 µg/ml; Sigma) or with proteinase K (ProtK; 40 µg/ml; Roche) as indicated, for 20 min at 37°C. Next, the trypsin inhibitor (5 µg/ml; Sigma) or the serine proteases inhibitor (PMSF 1 mM; Sigma) was added to stop further proteolysis. The cell suspension was centrifuged (14,000 xg, 1 min), the cell pellet resuspended in 50 µl of 10 mM Tris HCl pH 8.0, lysed with one volume of SDS-sample buffer (2X) or urea-SDS-sample buffer (2X), boiled and analyzed by Western blot.

### Purification of TirM_EHEC_

TirM_EHEC_ with a N-terminal His-tag was purified from 500 ml cultures of *E. coli* BL21(DE3) cells carrying pET28a-TirM_EHEC_ grown and induced as described above. Bacteria were lysed and the soluble protein extract loaded onto a chromatography column filled with a Cobalt-containing resin (Talon, Clontech). TirM_EHEC_ was eluted with 150 mM imidazole, dialyzed against HEPES-buffer (20 mM HEPES pH 7.4, 200 mM NaCl, sterile filtered and degassed), and loaded onto a calibrated gel filtration column (HiLoad 16/600 Superdex 75 preparative grade, GE Healthcare). Fractions containing TirM_EHEC_ were collected and checked for purity by SDS-PAGE. Protein concentration was estimated using the Bicinchoninic acid (BCA) Pierce protein assay kit (Thermo Scientific). Further details are described in Materials and Methods S1.

### Protein biotinylation

Biotinamidocaproate N-hydroxysuccinimide ester (Biotin-NHS; Sigma) was re-constituted at 25 mg/ml in dimethylsulfoxide (DMSO, Fluka) and immediately used. Purified protein (0.1-1 mg) [TirM_EHEC_, GFP (Upstate, Merck Millipore), BSA (Sigma), anti-E mAb (Phadia)] was mixed with Biotin-NHS (20-fold molar excess) in 1 ml of PBS and incubated for 2 h at RT with slow agitation on a gyratory wheel. The reaction was stopped by addition of Tris-HCl pH 7.5 at final concentration of 50 mM and the samples were placed on ice for 1 h. The reaction mix was loaded onto a pre-packed column for gel filtration chromatography (Sephadex G25 PD-10; GE Healthcare) and the biotinylated protein was eluted in 500-µl fractions with PBS. Protein concentration was estimated using the Bicinchoninic acid (BCA) Pierce protein assay kit (Thermo Scientific).

### Enzyme-linked immunosorbent assay (ELISA)

TirM_EHEC_ or BSA (Sigma) proteins were adsorbed at 4°C o/n onto 96-well immunoplates (Maxisorb; Nunc) at a concentration of 5 µg/ml in PBS. Next, immunoplates were washed in PBS and blocked by incubation with 200 µl of 3% (w/v) Milk -PBS for 2 h at RT. The sdAbs (secreted or purified) were diluted in 3% (w/v) Milk-PBS, added at the indicated concentrations (0.1-100 nM) in duplicates and incubated for 1 h at RT. After incubation, the wells were washed three times with PBS (Immunowash 1575, Bio-Rad) and the bound sdAbs was detected by the addition of anti-c-myc-POD mAb (clone 9E10; Roche; 1:1000), or anti-E-tag mAb (Phadia; 1:1000) followed by anti-mouse-POD (Sigma; 1:1000) for E-tagged sdAb, and incubation of the plates for 1 h at RT. The plates were washed three times with PBS and developed with H_2_O_2_ and o-phenylenediamine (OPD; Sigma) as previously described [38]. The plates were read at 490 nm using the iMark ELISA plate reader (Bio-Rad).

### Magnetic Cell Sorting (MACS)

Induced *E. coli* cells (equivalent to a final OD_600_ of 5.0) were harvested by centrifugation (4000 xg, 3 min), washed three times with 2 ml PBS (sterile filtered and degassed), and resuspended in a final volume of 1 ml of PBS. Biotinylated TirM_EHEC_ (at concentrations of 50 nM or 250 nM, as indicated) was added to 100 µl of bacteria, the final volume was adjusted to 200 µl with PBS-BSA (PBS supplemented with 0.5% w/v BSA, sterile filtered and degassed), and incubation was carried out for 1 h at RT. After incubation, bacteria were washed three times with 1 ml of PBS-BSA, resuspended in 100 µl of the same buffer containing 20 µl of anti-biotin paramagnetic beads (Miltenyi Biotec) and incubated at 4 °C for 20 min. Next, bacteria were washed three times with 1 ml of PBS-BSA, resuspended in 500 µl of the same buffer, of which 10 µl was kept aside to calculate the input bacteria before the procedure, while the rest (490 µl) was applied onto a MACS MS column (Miltenyi Biotec), previously equilibrated with 500 µl of PBS-BSA and placed on the OctoMACS Separator (Miltenyi Biotec). The flow through of unbound cells was collected and the column was washed three times with 500 µl of PBS-BSA. The wash was combined with the flow-through as “Unbound fraction”. Next, the column was removed from the OctoMACS Separator and placed onto a new collection tube, 2 ml of LB was added and the cells were eluted out. This fraction was labeled as the “Bound fraction”. Serial dilutions of Unbound and Bound fractions were plated to determine CFU and to harvest Bound bacteria.

### Flow cytometry analysis

For standard flow cytometry, induced bacterial cells (equivalent to a final OD_600_ of 1.0; ~10^9^ CFU) were harvested by centrifugation (4,000 xg, 3 min), washed twice with 500 µl of PBS (filter-sterilized) and resuspended in a final volume of 400 µl of PBS. Next, 190 µl of this cell suspension (~3x10^8^ CFU) was incubated with the primary antibody or antigen (as indicated) and PBS was added to adjust the total volume to 200 µl. The primary antibodies (for assay of expression levels) were anti-E-tag mAb (1:200; Phadia) or anti-c-myc mAb (1:200; 9B11 clone; Cell Signalling), while biotinylated antigens (GFP, TirM_EHEC_, BSA) were used at 50 nM for assay of antigen binding, unless otherwise indicated. The samples were incubated at RT for 1h. After incubation, the cells were washed once with 500 µl of PBS, and resuspended either in 500 µl of PBS containing 1 µl of anti-mouse-IgG1 conjugated to Alexa 488 Fluor (2 mg/ml, Invitrogen) or in 200 µl of PBS containing 30 µl of 1:200 dilution of Streptavidin-phycoerythrin (PE) (0.5 mg/ml, Beckman Coulter). The mixture was incubated 30 min at 4 °C in the dark. The cells were washed once with 500 µl of PBS and resuspended in a final volume of 1 ml in PBS. For each experiment at least 100,000 cells were analyzed in a cytometer (Gallios, Beckman Coulter).

### Affinity determination by flow cytometry analysis

Induced *E. coli* cells (equivalent to final OD_600_ of 1) were centrifuged (4000 xg, 3 min), washed twice with 1 ml of PBS (filter-sterilized) and resuspended in a final volume of 1 ml of PBS. Next, 50 µl of this cell suspension (~3x10^7^ CFU) was incubated at room temperature for 90 min with a fixed amount of biotinylated TirM_EHEC_ (2 pmols; ~30 ng) and increasing volumes of PBS (from 0.1 to 1.5 ml) to attain a final concentration range between 20 nM to 1 nM. After incubation, cells were centrifuged (4000 xg, 3 min), washed twice with 1 ml of PBS (filter-sterilized) and labeled with Streptavidin-PE as described for standard flow cytometry. After a final washing step with PBS, the mean fluorescence intensity (MFI) of Phycoerythrin (PE) was quantified in a cytometer (Gallios, Beckman Coulter). Data of MFI (relative values to maximum MFI) obtained from the cytometer were plotted against the concentration of TirM_EHEC_ to obtain the dissociation constant (K_D_). Curve was fitted according to non-linear least squares regression method and one site - specific binding saturation kinetics model using the data analysis tool in Prism software (GraphPad).

### Secretion of sdAbs into *E. coli* culture media

Secretion of Ab fragmemts to *E. coli* culture media with the hemolysin secretion system has been reported previously [34,35]. Induced cultures of 10 ml *E. coli* HB2151 cells transformed with pVDL9.3 and pEHlyA4SD-V_HH_ derivative were centrifuged (10000 xg, 10 min, 4°C) and the culture supernatants were adjusted to PBS 1X by adding 1/10th volume of PBS 10X. The amount of secreted V_HH_-HlyA fusion (~5 µg/ml) was estimated by densitometric analysis of silver-stained SDS-polyacrylamide gels loaded with 10 µl samples of culture supernatants and dilutions of BSA standards of known concentration (Thermo Scientific). When needed, the V_HH_-HlyA fusions in culture supernatants were concentrated with 3-kDa or 10-kDa centrifugal filter units (Amicon Ultra-15).

### Purification of sdAbs from the periplasm of *E. coli*

Soluble sdAbs with hexahistidine and myc tags in their C-termini were induced in *E. coli* WK6 cells carrying pCANTAB6-VTIR1 or pCANTAB6-Vgfp. Cells were pelleted by centrifugation (4000 xg, 12 min, 4°C) from 1 L cultures, resuspended in 22.5 ml Periplasmic Extraction buffer [50 mM Sodium phosphate pH 7.4, 200 mM NaCl, 5 mM EDTA and 1 mg/ml polymyxin B sulphate (Sigma)] and stirred at 4°C for 2 h using a magnetic stirrer. The periplasmic extract was obtained by ultracentrifugation (40000 xg, 30 min, 4°C) and dialyzed o/n at 4°C against 5 L of PN2 buffer (50 mM sodium phosphate pH 7.4, 200 mM NaCl). Dialyzed extract was loaded onto a Cobalt-containing affinity resin (Talon, Clontech), washed, and bound protein eluted in PN2 with 150 mM imidazole. Eluted sdAb was dialyzed, concentrated, and loaded onto a calibrated gel filtration column (HiLoad 16/600 Superdex 75 preparative grade, GE Healthcare) as described previously for TirM_EHEC_. The fractions corresponding to the monomeric sdAb were collected and concentrated in a 3-kDa centrifugal filter unit (Amicon Ultra-15). Protein concentration was estimated using the Bicinchoninic acid (BCA) Pierce protein assay kit (Thermo Scientific).

### Surface Plasmon Resonance affinity determination

SPR measurements were performed using a Biacore 3000 instrument (GE Healthcare). All proteins solutions were dialyzed against HEPES-buffer [20 mM HEPES 200 mM NaCl (pH 7.4) sterile filtered and degassed] at 4°C o/n. Biotinylated TirM_EHEC_ (0.1 µg/ml) was immobilized on a Streptavidin SA chip (GE Healthcare) at 150 response units (RU) at a flow rate of 10 µl/min in HEPES-buffer containing 0.005% (v/v) of the surfactant Polysorbate 20 (P20, GE Healthcare). For determination of binding kinetics, dilutions of purified sdAb (analyte) from 32 nM to 200 pM were flown at 30 µl/min in HEPES-buffer and sensograms were generated. The biotinylated TirM_EHEC_ surface on the Streptavidin SA chip was regenerated after every cycle using three injections of 10 µl of 10 mM Glycine-HCl (pH 1.7). Sensograms with different concentrations of anaylte were overlaid, aligned and analyzed with BIAevaluation 4.1 software (GE Healthcare). All data were processed using a double-referencing method [39].

## Results

### Comparison of EhaA and Intimin β-domains for display of sdAbs on *E. coli*

To test the potential of Intimin β-domain for the display of Ab fragments and compare it with the display capacity of EhaA β-domain, we employed sdAbs derived from heavy-chain antibodies (HCAbs) of camelids (e.g. dromedaries, llamas) also known as VHH (for VH of HCAbs) or nanobodies [40]. These sdAbs have a small size (ca. 12-14 kDa), high stability and solubility, as well as excellent antigen binding properties with high affinity and specificity. In addition, their sequence identity with human VH makes them attractive for therapy [41]. Initial assays were conducted using a model VHH clone binding GFP (Vgfp) [42] fused to the β-domains of EhaA and Intimin for comparison of the two systems before cloning of an immune library of sdAbs. Vgfp was cloned in pHEA vector (Table 1) in frame with the N-terminal SP of PelB [43] and the C-terminal fragment of EhaA (residues 989-1327; named as C-EhaA), bearing its native β-barrel with α-helix linker, and including the E-tag epitope between the VHH and C-EhaA (Figure 1A) [33]. The Vgfp sequence was cloned in pNeae2 (Table 1) in frame with the N-terminal fragment of Intimin (residues 1-659; named as Neae), comprising its N-terminal SP, the periplasmic LysM domain (expected to bind the peptidoglycan), its native β-barrel with C-terminal linker, and the first Ig-like domain (D0) (Figure 1B). Vector pNeae2 incorporated the E-tag and myc-tag epitopes flanking the VHH (Figure 1B). Both *E. coli* display vectors contained unique *Sfi*I and *Not*I restriction sites flanking the VHH in the same frame as those of conventional phagemids (e.g. pHEN6, pCANTAB6) [44]. The resulting fusion proteins were referred to as VHHA (fusions to C-EhaA) and NVHH (fusions to Neae).

The expression of VgfpA and NVgfp fusions in *E. coli* K-12 cells (strain UT5600; Table 1) was analyzed by Western blot after induction with 0.05 mM IPTG at 30 °C for 3 h (see Materials and Methods). Discrete protein bands corresponding to VgfpA and NVgfp were detected with anti-E or anti-myc monoclonal Abs (mAbs) in whole cell protein extracts from the induced cells (Figure 1C and Figure 1D). Both fusion proteins showed a shift in their electrophoretic mobility, characteristic of native OMPs with correctly folded β-barrels, which are resistant to SDS denaturation at low temperatures and hence migrate faster than the unfolded polypeptides due to the compact structure of the β-barrel [17,45]. This heat-modifiable mobility was observed in both VgfpA and NVgfp, having the expected mobility according to their molecular weight (MW) after boiling (ca. 52 kDa and 84 kDa, respectively) and a faster mobility in non-boiled samples (Figure 1C and 1D). Interestingly, NVgfp was resistant to 2% SDS and 4 M urea at low temperatures (i.e. 22 °C) and required boiling in this buffer to unfold, as previously reported for full-length Intimin and its β-domain [25]. The major protein bands detected with anti-E mAb in the boiled samples corresponded to full-length VgfpA and NVgfp fusions (Figure 1C and 1D; labeled with arrows). Detection of NVgfp with anti-myc mAb confirmed the integrity of its C-terminal end (Figure 1D). Minor bands of lower MW were also detected in Western blot with anti-E mAb, which likely represent proteolytic fragments of the full-length fusions (Figure 1C and 1D; labeled with asterisks). The induced *E. coli* cultures expressing VgfpA or NVgfp fusions showed only a slight decrease in their growth rate compared to control cultures having the empty vector (pAK-Not), or expressing the β-domains C-EhaA (pHEA) or Neae (pNeae2), and reached final optical densities at 600 nm (OD_600_) identical to controls (Figure S1).

The accessibility of VgfpA and NVgfp fusions to the external milieu was initially compared by incubation of intact *E. coli* cells with externally added proteases. Trypsin digested full-length VgfpA leaving some weakly detectable proteolytic fragments with a size similar to C-EhaA and Vgfp domains (Figure 1C, lane 3). The NVgfp fusion was resistant to Trypsin digestion (Figure S2) but was sensitive to Proteinase K (ProtK) (Figure 1D, lane 3). Nevertheless, ProtK digestion of NVgfp fusion left a resistant fragment comprising Neae. Hence, both the β-domains display the sdAb to the extracellular milieu, in a way that is accessible to externally added proteases but C-EhaA fusions are more sensitive to digestion than Neae fusions. Resistance to proteolysis was previously observed for full-length Intimin [25].

**Figure 2:**
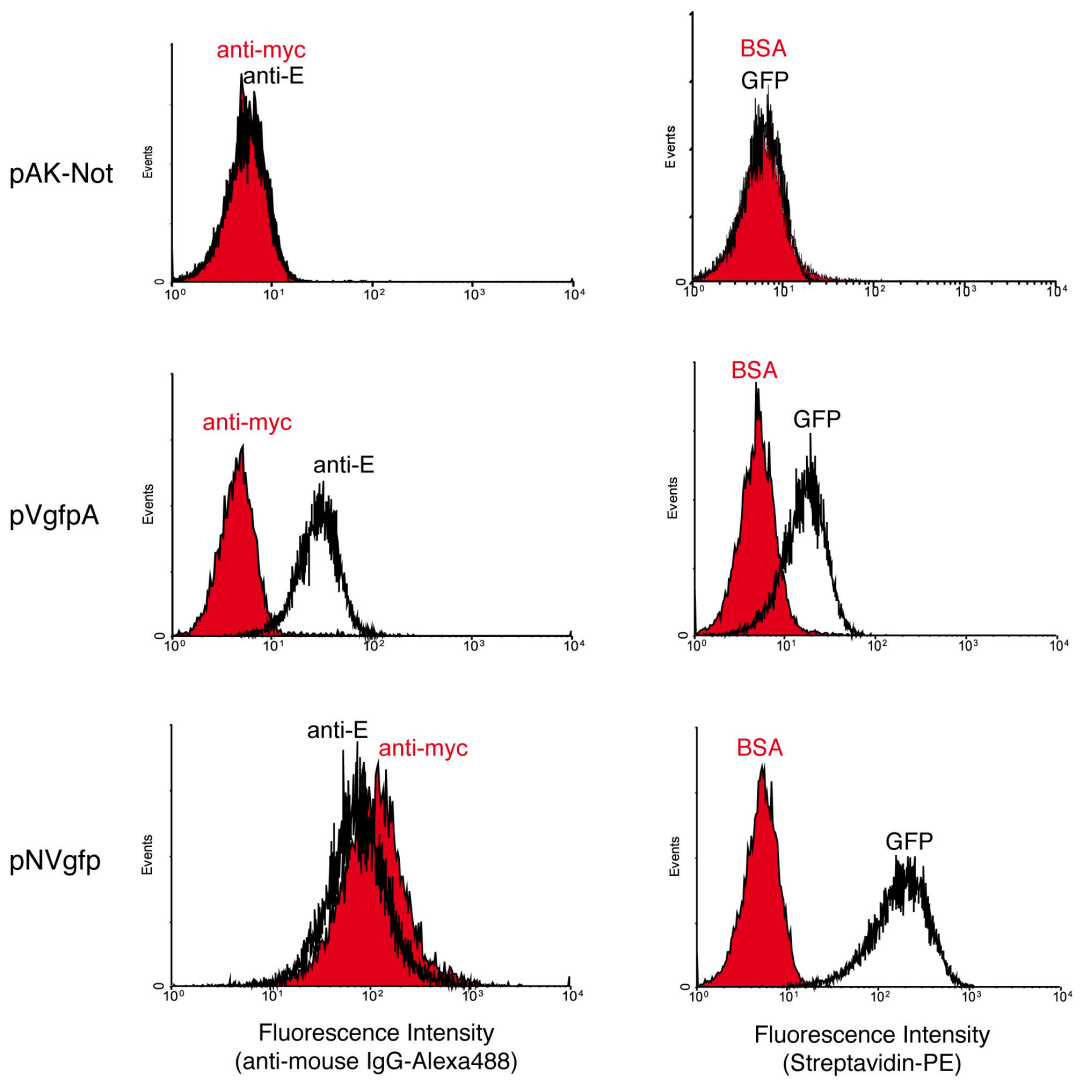
*E. coli* cell surface display and antigen binding activity of VgfpA and NVgfp fusions. Fluorescent flow cytometry analysis of induced *E. coli* UT5600 cells bearing the indicated plasmids: pAK-Not (control), pVgfpA, and pNVgfp. Histograms show the fluorescence intensity of bacteria stained with anti-E or anti-myc mAbs (as indicated) and secondary anti-mouse IgG-Alexa 488 (left panels) or incubated with biotinylated antigens (GFP or BSA, as labeled) and secondary Streptavidin-phycoerythrin (PE) (right panels).

Surface display of VgfpA and NVgfp was assessed by flow cytometry (Figure 2). Induced *E. coli* cells harboring pVgfpA, pNVgfp, or pAK-Not (control) were stained with anti-E or anti-myc mAbs followed by anti-mouse IgG-Alexa488 (Figure 2, left panels). *E. coli* cells expressing VgfpA or NVgfp were positively bound by anti-E mAb, though cells expressing NVgfp were also positively bound with the anti-myc mAb. Control *E. coli* cells with pAK-Not were negative for both mAbs. Importantly, the presence of a single peak in the flow cytometry histograms indicated that most *E. coli* cells were expressing a homogenous level of the fusion proteins. The mean fluorescence intensity (MFI) of cells with anti-E-tag mAb suggested a higher expression and display level of NVgfp than VgfpA (~3-fold).

The antigen-binding activity of the surface displayed Vgfp sdAb using both display systems was compared by flow cytometry after incubation of *E. coli* cells expressing the fusions with 50 nM biotin-labeled GFP (positive antigen) or biotin-labeled BSA (negative antigen), followed by incubation with Streptavidin-Phycoerythrin (PE) conjugate (Streptavidin-PE) (Figure 2, right panels). This analysis showed the specific binding of the *E. coli* cells expressing VgfpA or NVgfp fusions to GFP, whereas control *E. coli* cells did not bind GFP. None of these cells bound BSA. Therefore, the sdAb is functional when displayed on the surface of *E. coli* cells with the β-domains of EhaA and Intimin. Nevetheless, the MFI of GFP binding was higher in *E. coli* cells expressing NVgfp than in those with VgfpA (ca. 8-fold) (Figure 2, right panels). The 3-fold difference in expression level of NVgfp does not appear to be sufficient to account for this difference in binding, suggesting that the sdAb may have a higher antigen-binding activity when fused to the β-domain of Intimin (see Discussion).

### Selection of antigen binding sdAbs from an immune library displayed on *E. coli* cells

To test the effectiveness of the *E. coli* display systems for the expression of a VHH library and selection of antigen-binding clones, an immune library against the soluble extracellular fragment of the translocated intimin receptor (tir) from EHEC (named TirM_EHEC_, corresponding to residues 252-360 of full-length Tir_EHEC_) [46], was generated and cloned in vectors pHEA and pNeae2. TirM_EHEC_ binds to the C-terminal Ig-like and lectin-like domains (D2-D3) of full-length Intimin_EHEC_ [30,47] but not to the β-domain of Intimin (Neae) used for in this study. The VHH library against TirM_EHEC_ was obtained by immunization of a dromedary with purified recombinant his-tagged TirM_EHEC_ and subsequent amplification of the VHH gene segments from ~2x10^7^ lymphocytes isolated from a peripheral blood sample (Materials and Methods). The amplified VHH gene segments were cloned into the *Sfi*I and *Not*I sites of pHEA and pNeae2 vectors, generating two *E. coli* display immune libraries of similar size (~2-3x10^6^ clones). This relatively small library size is reported to be sufficient for having a good representation of the repertoire of V_HH_ genes in the peripheral blood of camelids after immunization and to select the sdAbs of higher affinity raised against the antigen using conventional phage display [48]. The *E. coli* strain EcM1 (Table 1) was used as host for cell display. This strain is derived from the reference wild type K-12 strain (MG1655) with a deletion in the operon encoding type 1 fimbriae (Δ*fim*A-H) [49]. Sequencing of 40 clones picked randomly from each library confirmed the cloning of different VHH sequences in frame with the β-domains of EhaA and Intimin (data not shown).

**Figure 3:**
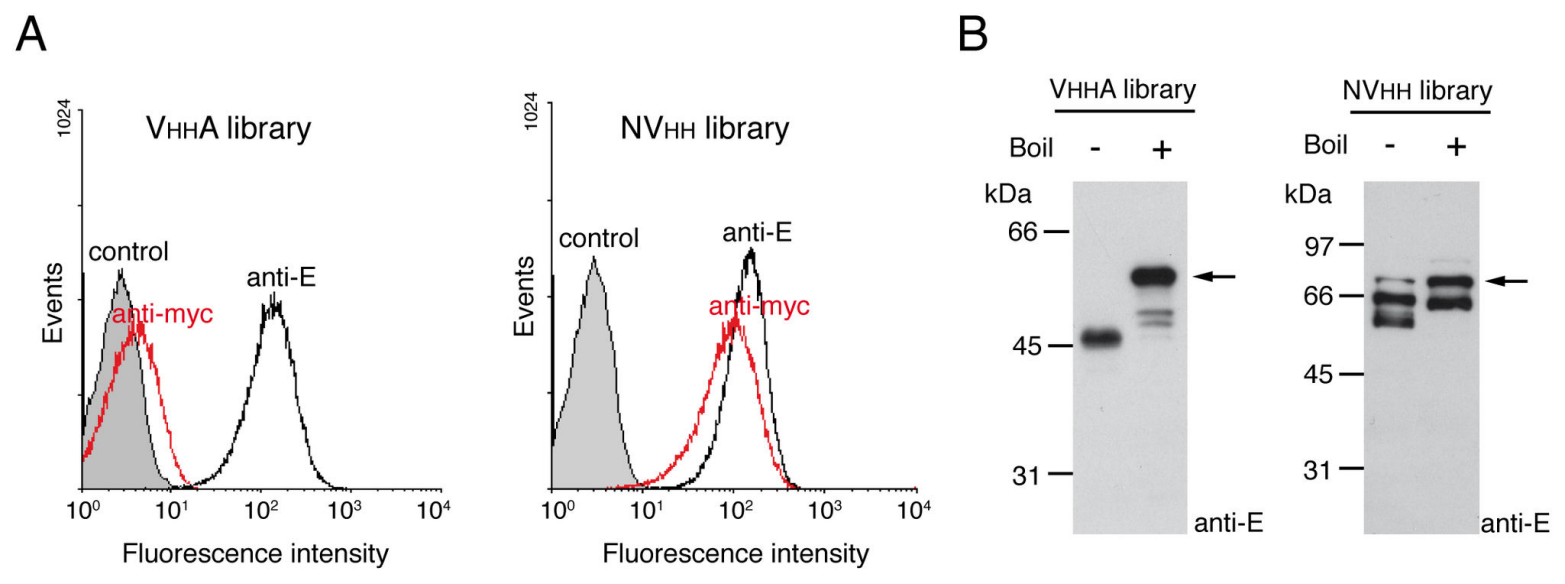
*E. coli* cell surface display of VHHA and NVHH immune libraries. (**A**) Fluorescent flow cytometry analysis of induced *E. coli* EcM1 cells expressing VHHA or NVHH immune libraries anti-TirM_EHEC_ (as indicated). Control cells carried the empty vector pAK-Not. Histograms show the fluorescence intensity of bacteria stained with anti-E or anti-myc mAbs (as labeled) and secondary anti-mouse IgG-Alexa 488. (**B**) Western blots of whole-cell protein extracts from induced *E. coli* EcM1 cells expressing VHHA or NVHH immune libraries anti-TirM_EHEC_ (as indicated). Protein extracts were prepared in SDS (VHHA library) or SDS-urea (NVHH library) sample buffers and boiled (+) or not boiled (-) before SDS-PAGE. Western blots were developed with anti-E mAb. The positions of full-length fusions are labeled with arrows. The mass of protein markers (in kDa) is shown on the left.

The expression and display of the VHH libraries with the β-domains of EhaA (VHHA) and Intimin (NVHH) were analyzed by flow cytometry with anti-E and anti-myc mAbs, revealing a fairly homogeneous expression of both libraries in *E. coli* EcM1 (Figure 3A). The MFI with anti-E mAb indicated a similar expression level of VHHA and NVHH libraries, in contrast to the significantly lower expression of VgfpA observed previously. Western blot analysis of whole-cell protein extracts from induced cultures revealed major protein bands with the expected size for full-length VHHA and NVHH fusions, upon boiling in SDS or SDS-urea buffer, respectively, and which have heat-modifiable electrophoretic mobility indicating the correct folding of their β-barrels (Figure 3B). Quantification of the Western blot signals with anti-E mAb using ~1.5x10^8^ bacteria (0.15 units of OD_600_) expressing VHHA or NVHH fusions, was carried out using a standard curve generated with a purified E-tagged VHH of known concentration (Figure S3), and allowed an estimation of ~6.5x10^3^ molecules of VHHA and ~7.8x10^3^ molecules of NVHH per bacterium.

The growth of *E. coli* cultures expressing VHHA or NVHH libraries was only slightly delayed compared to a control with pAK-Not and the cultures reached similar OD_600_ after induction (Figure S4). Plating of the induced cultures to determine the number of colony forming units (CFU) per OD_600_ gave ~1.0x10^9^ CFU/OD_600_ in the control, ~ 0.9 x10^9^ CFU/OD_600_ in the VHHA library, and ~0.6x10^9^ CFU/OD_600_ in the NVHH library. Hence, although growth is not affected, expression of NVHH fusions appear to reduce the viability of *E. coli* cells compared to control cultures. Nevertheless, since the CFU/OD_600_ after expression of NVHH fusions remains within the same order of magnitude as the control, the diversity of the sdAb library is not compromised. Cell toxicity for the expression of Intimin constructs has been reported previously in some *E. coli* K-12 strains [22].

**Figure 4:**
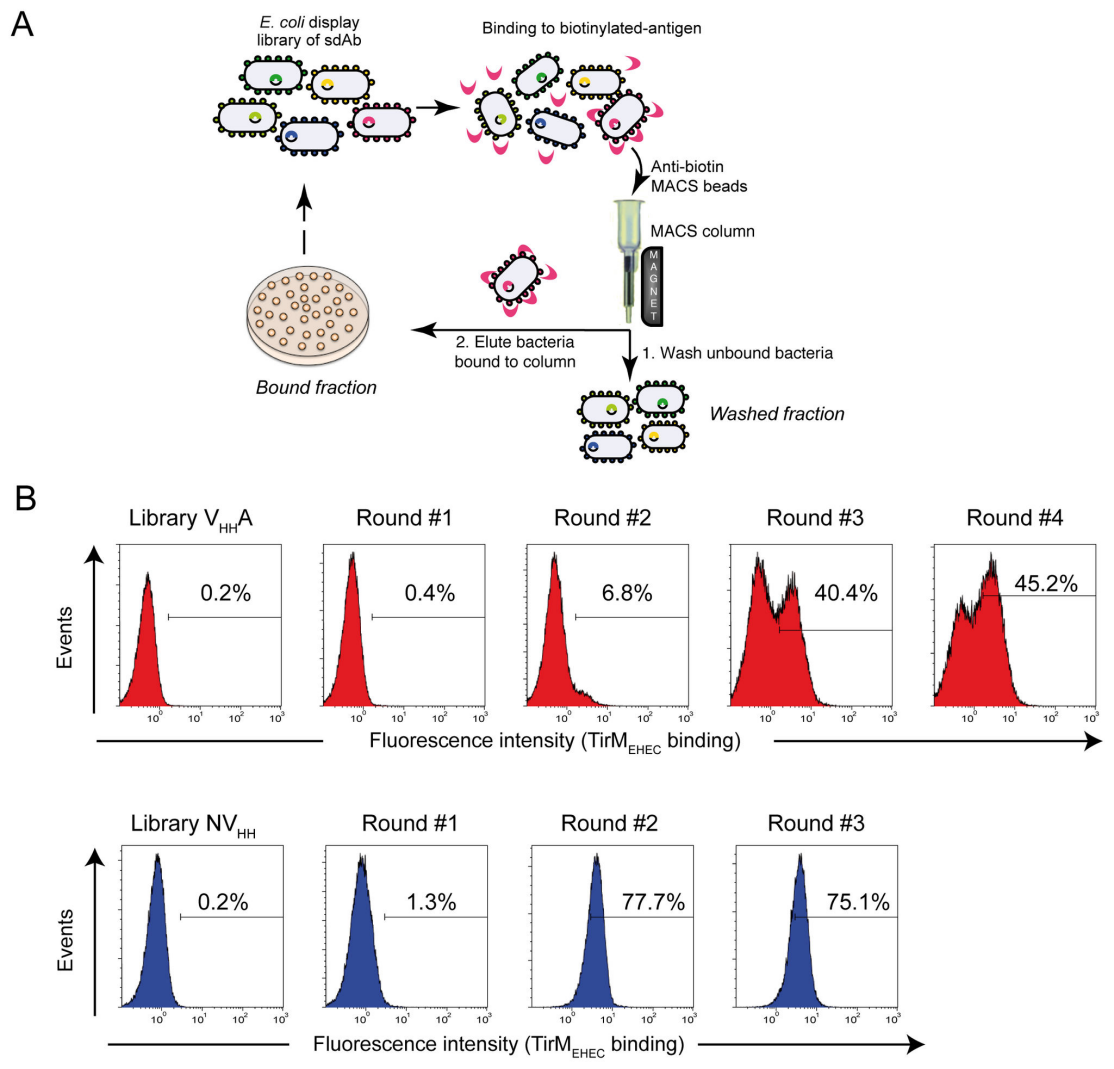
Magnetic cell sorting of *E. coli* display libraries VHHA and NVHH with biotinylated antigen TirM_EHEC_. (**A**) General scheme summarizing the steps followed during MACS of an *E. coli* display library of sdAb with a biotinylated antigen. *E. coli* cells binding the biotinylated antigen are captured in a MACS column held in a magnet, while *E. coli* cells that do not bind the antigen are washed out of the column. Elution of bound bacteria is done with fresh LB media upon column removal from the magnet. The CFU in the Washed and Bound fractions are determined by plating. (**B**) Fluorescent flow cytometry analysis of IPTG-induced *E. coli* EcM1 cells expressing VHHA (top panel) or NVHH (bottom panel) immune libraries, or their respective sublibraries enriched after the indicated round of MACS with biotinylated TirM_EHEC_. Histograms show the fluorescence intensity of bacteria incubated with biotinylated TirM_EHEC_ and secondary Streptavidin-PE.

*E. coli* EcM1 cells expressing VHHA and NVHH libraries were screened to isolate clones binding to TirM_EHEC_ by magnetic cell sorting (MACS). In MACS, cells are incubated with a biotinylated target protein (e.g. antigen, mAb, etc.), washed to remove the unbound protein, and incubated with a suspension of paramagnetic microbeads coupled to Streptavidin or a mAb binding biotin (anti-biotin microbeads). The cell mixture is then passed through a small ferromagnetic column held in a magnetic holder, which retains cells coated with anti-biotin microbeads whereas unbound cells are washed out of the column (Figure 4A). Elution of bound cells is carried out with buffer or fresh culture media (e.g. LB) upon removal of the column from the magnet. The CFU washed out of the column (Washed fraction) and eluted (Bound fraction) are determined by plating. The MACS conditions for capturing *E. coli* cells expressing VHHA or NVHH fusions were established using biotin-labeled anti-E mAb. An initial input of 0.1 units of OD_600_ (~6-9x10^7^ CFU of each culture) was incubated with 50-250 nM of biotinylated anti-E mAb allowing the recovery of a total of ~2-4x10^7^ CFU in the Washed and Bound fractions. The Bound fractions contained ~95-99% of the CFU in the VHHA and NVHH libraries, and only ~0.2-0.5% in control *E. coli* cells with pAK-Not. Using these conditions, the VHHA and NVHH libraries were incubated with biotinylated TirM_EHEC_ (250 nM) for the first selection step (MACS1) and ~0.3-0.6% of the total CFU from both libraries were collected in the Bound fractions (Table 2). The colonies grown from the Bound fractions of each library were pooled independently and their plasmids purified and electroporated into fresh *E. coli* EcM1 cells to obtain VHHA and NVHH sublibraries (≥2x10^6^ transformants). This step was added to avoid multiple rounds of induction of the same bacterium, which may favor the amplification of clones with reduced levels of expression of the fusions.

**Table 2:** Summary of MACS with *E. coli* display libraries.

Round	TirM_EHEC_ (nM)	VHHA library (%)	NVHH library (%)
MACS1	250	0.3	0.6
MACS2	250	2.6	12.5
MACS3	50	12.5	75.6
MACS4	50	70.5	ND

(%) : Percentage of bacteria in Bound fractions with reference to total bacteria in Wash+Bound fractions (ca. 1-5 x 10^7^ CFU)

Next, the VHHA and NVHH sublibraries were subjected to a new round of selection with biotinylated TirM_EHEC_ using conditions identical to those used in MACS1. Bacteria harvested from Bound fractions were pooled, their plasmids purified and transformed for the following rounds of MACS. Antigen concentration was reduced to 50 nM in the following MACS. The percentage of *E. coli* bacteria recovered in the Bound fractions showed a significant increase from the initial 0.3-0.6% to over 70% in MACS3 of NVHH and MACS4 of VHHA (Table 2), suggesting an enrichment of antigen binding clones in both libraries. Bacteria from the different rounds of selection were analyzed by flow cytometry to test their binding to biotinylated TirM_EHEC_ (50 nM) (Figure 4B), which demonstrated an enrichment of *E. coli* cells binding to TirM_EHEC_ along the selection rounds, from ~0.2% positives in the original libraries to ~45% after MACS4 of the VHHA library and more than 75% positives after MACS2 and MACS3 of the NVHH library. No significant binding to biotinylated BSA was detected by flow cytometry in these pools (data not shown). The expression levels of the VHHA and NVHH fusions in the bacterial pools obtained after MACS was similar to those of the original libraries (data not shown).

**Figure 5:**
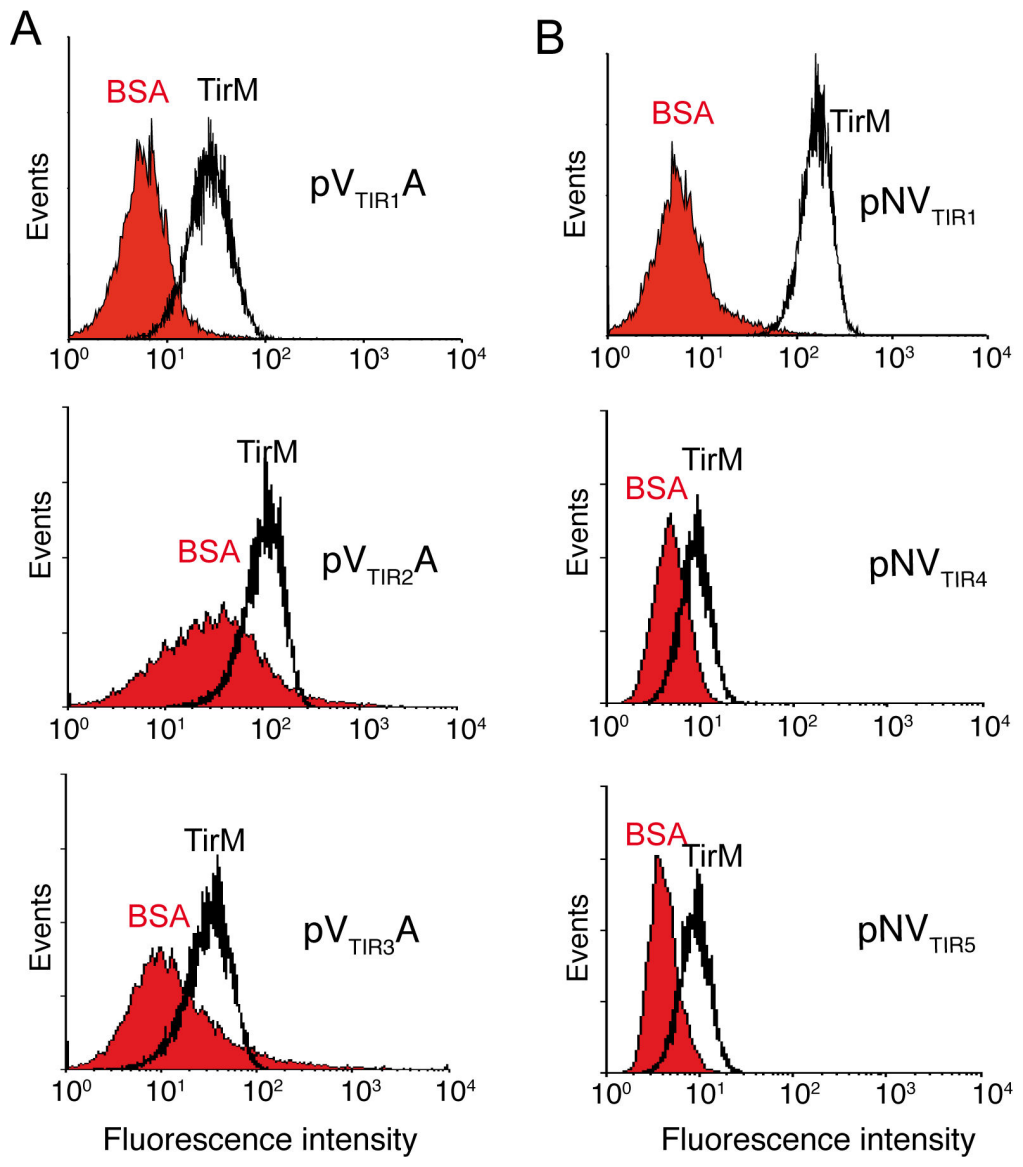
Binding to TirM_EHEC_ by *E. coli* cells displaying selected clones from VHHA and NVHH libraries. Fluorescent flow cytometry analysis of induced *E. coli* EcM1 cells bearing the indicated plasmids selected from (**A**) the VHHA library: pVTIR1A, pVTIR2A, pVTIR3A; and (**B**) from the NVHH library: pNVTIR1, pNVTIR4, pNVTIR5. Histograms show the fluorescence intensity of bacteria incubated with biotinylated antigens (TirM_EHEC_ or BSA, as labeled) and secondary Streptavidin-PE.

**Figure 6:**
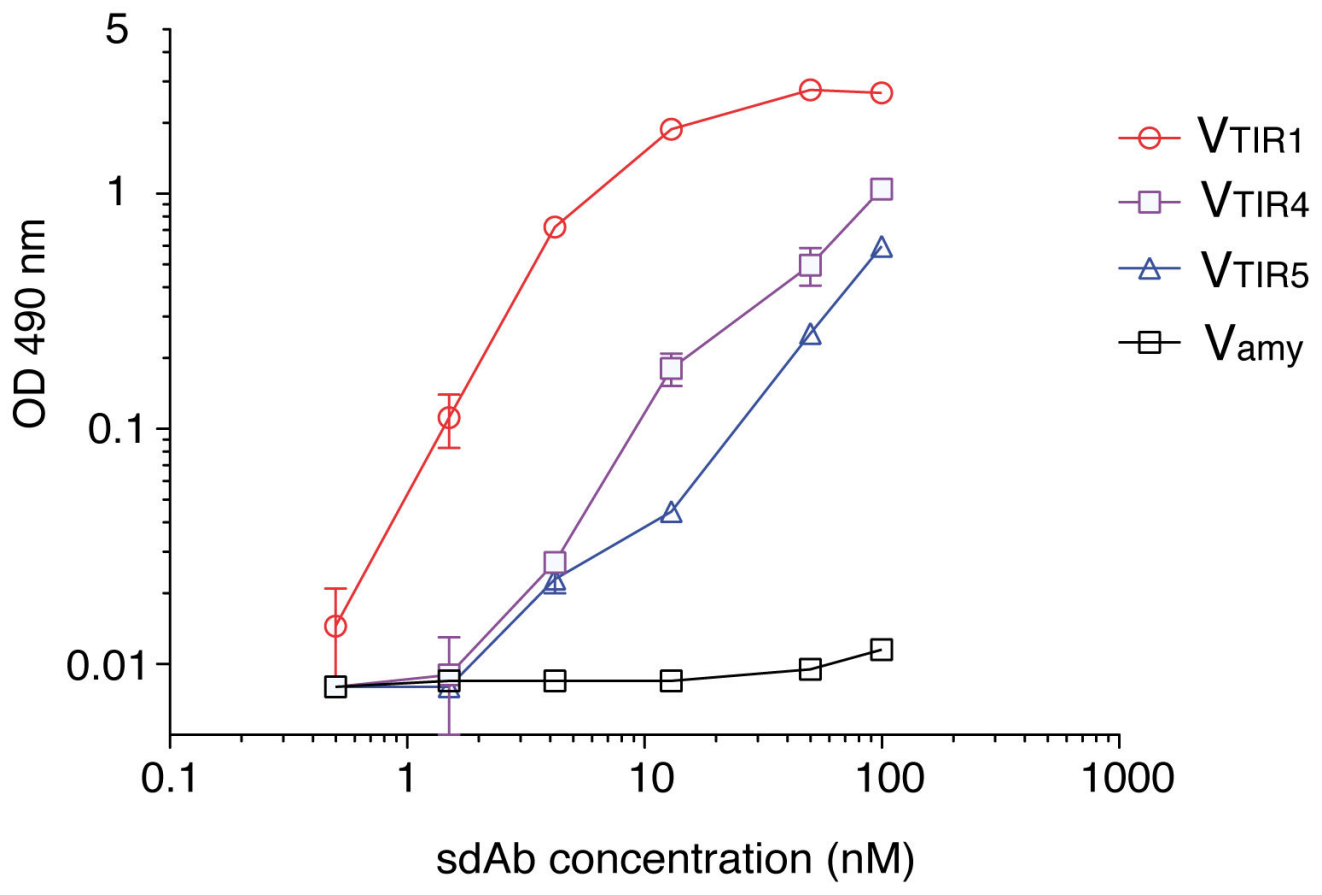
ELISA of sdAbs selected by *E. coli* display against TirM_EHEC_. ELISA against TirM_EHEC_ of sdAbs secreted into culture media as E-tagged HlyA fusions from the indicated VTIR clones and one a negative control (Vamy) [35]. The plot shows the average OD values at 490 nm with standard error from duplicate experimental samples obtained with the secreted sdAbs at the indicated concentrations. ELISA were developed with anti-E-tag mAb and anti-mouse-POD. ELISA signals against a control antigen (BSA) are subtracted from the represented values.

Fifty colonies from the final round of selection of each library were randomly picked for plasmid isolation and DNA sequencing. A VHH sequence, named as VTIR1, was found in all NVHH clones and in 36 VHHA clones, the rest being different VHH sequences. Flow cytometry analysis confirmed the specific binding of biotinylated TirM_EHEC_ (50 nM) by *E. coli* cells displaying VTIR1 fused to EhaA and Intimin β-domains whereas these cells did not bind to biotinylated BSA (Figure 5A and 5B). Similar to the situation observed with Vgfp clone, the MFI of *E. coli* cells displaying VTIR1 was higher in the NVTIR1 fusion than with VTIR1A fusion, although both were expressed at similar levels (Figure S5). We conducted a flow cytometry screening of the remaining 14 non-VTIR1 clones from MACS4 of VHHA library, identifying three other VHH sequences that bound to biotinylated TirM_EHEC_, referred to as VTIR2, VTIR3, VTIR4 (Figure 5A, and data not shown). VTIR4 did not bind biotinylated BSA, but VTIR2 and VTIR3 clones exhibited non-specific binding to biotinylated BSA and were not further analyzed (Figure 5A and 5B). We sought for additional VHH sequences binding TirM_EHEC_ in the NVHH library by screening 96 colonies picked randomly after the first round of selection (MACS1), in which a higher diversity of binders with low and high affinities could be expected. PCR screening with a specific primer hybridizing the complementarity determining region 3 (CDR3) of VTIR1 enabled us to identify 17 VTIR1 clones out of these 96 colonies. This number is higher than the 1.3% positives found by flow cytometry after MACS1 of the NVHH library (Figure 4B) but fits better with the percentage of clones recovered from this population after MACS2 (Table 2). This could indicate a lower sensitivity of flow cytometry to actually discriminate positive binders from the population. Flow cytometry screening of the remaining clones allowed the identification of two additional VHH sequences that specifically bound biotinylated TirM_EHEC_, VTIR4 (2 clones), which was found previously in the VHHA library, and VTIR5 (1 clone) (Figure 5B). The MFI of these clones with 50 nM biotinylated TirM_EHEC_ was low compared to VTIR1 (Figure 5B) and increased at higher antigen concentrations (200 nM) (data not shown) suggesting that these clones had a lower affinity for TirM_EHEC_. The CDR3 amino acid sequences of the selected VHH are shown in Table 3. The sdAbs encoded by VTIR1, VTIR4, VTIR5 and one unrelated V_HH_ binding α-amylase as a control, were secreted into *E. coli* culture media as soluble fragments with the hemolysin system [34,35], and used in ELISA plates coated with TirM_EHEC_ and BSA (Materials and Methods). This experiment showed that soluble VTIR1, VTIR4, and VTIR5 sdAbs, lacking the β-domains of EhaA and Intimin, also bound specifically to TirM_EHEC_ (Figure 6). VTIR1 was the clone with an apparent higher affinity, as could be inferred from its enrichment in both the *E. coli* display libraries.

**Table 3:** VHH clones selected against TirM_EHEC_ by *E. coli* display.

**Clone name**	**Amino acid sequence of CDR3**	**β-domain system**
V_TIR1_	GTAPYWHTPIPTLSEDKYFY	Neae, C-EhaA
V_TIR2_	GNSGSRGFDY	C-EhaA
V_TIR3_	AKGPRRCNQGFDY	C-EhaA
V_TIR4_	PDLSTNCDTVLTNSGALYNY	Neae, C-EhaA
V_TIR5_	PKYGGTWRWRVEEEKTTI	Neae

### Characterization of VTIR1 sdAb and determination of its affinity by surface plasmon resonance and *E. coli* display

**Figure 7:**
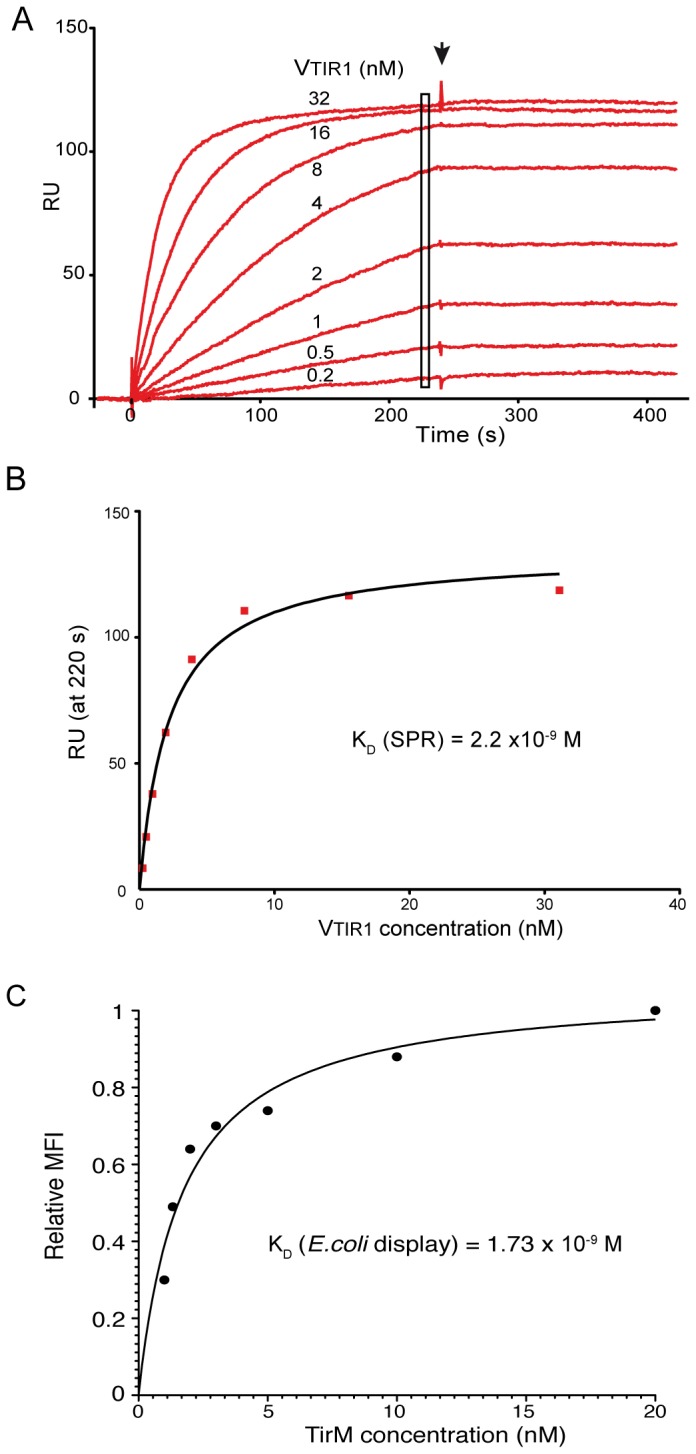
Determination of the equilibrium dissociation constant (K_D_) of sdAb VTIR1 by SPR and *E. coli* display. (**A**) SPR sensograms monitoring real-time association and dissociation of purified sdAb VTIR1 (at the indicated concentrations) to biotinylated TirM_EHEC_ immobilized onto a Streptavidin-SA sensor chip. The increase in resonance units (RU) is recorded along time (in seconds). Dissociation of VTIR1 is evaluated by injection of buffer at the time indicated with an arrow. (**B**) RU values at 220 seconds (labeled with a rectangle in A) are plotted *versus* the different concentrations of VTIR1. The curve was fitted by non-linear least squares regression. (**C**) The K_D_ of VTIR1 was estimated by flow cytometry analysis of *E. coli* cells expressing NVTIR1 incubated with different concentrations of biotinylated TirM_EHEC_ (1-20 nM) under equilibrium conditions. The mean fluorescent intensities (MFI) of bacteria, after labeling with Streptavidin-PE, were plotted *versus* the concentration of TirM_EHEC_ used in the assays. The curve was fitted by non-linear least squares regression.

The VTIR1 clone was produced in the periplasm of *E. coli* WK6 cells as soluble sdAb with C-terminal His- and myc-tags and purified by metal-affinity chromatography followed by gel-filtration chromatography (Materials and Methods). As a control, Vgfp was also expressed and purified in the same manner. Both sdAbs behave as monomers with an apparent mass of ~15 kDa in gel filtration chromatography (Figure S6A). The binding activity of the purified VTIR1 was confirmed in ELISA (Figure S6B). In order to determine the apparent equilibrium dissociation constant (K_D_) between VTIR1 and TirM_EHEC_ their interaction was studied in surface plasmon resonance (SPR) experiments with a Streptavidin (SA) sensor chip coated with biotinylated TirM_EHEC_ (Materials and Methods). The change in resonance units (RU) was recorded with time at different concentrations of purified VTIR1 from 0.2 to 32 nM showing a clear binding to TirM_EHEC_ that reached the steady state equilibrium in ~220 s for the two highest concentrations used, but not for the lower concentrations (Figure 7A). No binding was observed when Vgfp (40 nM) was flown over this sensor surface, or when VTIR1 (40 nM) was flown over a SA flow cell lacking biotinylated TirM_EHEC_ (data not shown). Injection of buffer to evaluate the dissociation of VTIR1 (labeled with an arrow in Figure 7A) showed no loss of RU for > 200 s, indicating that VTIR1 remained stably bound to TirM_EHEC_ over long periods of time. The absence of dissociation of VTIR1 from TirM_EHEC_ prevented determination of its kinetic constants (*k*_on_ and *k*_off_) from the sensograms. Since reaching the steady state at the lower concentrations of VTIR1 would require very long injection times (>1 h), which are not practical due to the higher amounts of Ab needed and the possibility of conformational changes of the antigen bound to the sensor chip, in order to have an estimation of the apparent K_D_ between VTIR1 and TirM_EHEC_ we plotted the RU values obtained with the different concentrations of VTIR1 at 220 s, the time at which two concentrations had reached the steady state. This plot provided an apparent K_D_ of ~2.2 x 10^-9^ M (Figure 7B). Nevertheless, the actual K_D_ between VTIR1 and TirM_EHEC_ should be below this estimated value (indicating an even higher affinity of VTIR1 for TirM_EHEC_) since the steady state equilibrium has not been reached at the lower concentrations.

Flow cytometry analysis under equilibrium conditions has been used to estimate the apparent K_D_ of Abs and Anticalins displayed on the surface of yeast and *E. coli* cells [14,23,50]. Thus, we tested whether the affinity of VTIR1 could also be estimated by flow cytometry analysis of *E. coli* cells with this sdAb on their surface and incubated with biotinylated TirM_EHEC_ under conditions expected to be close to the equilibrium. We chose the Intimin display system given its superior MFI signals in flow cytometry with the antigen. *E. coli* EcM1 cells displaying NVTIR1 (~3x10^7^ CFU) were incubated for 90 min with a fixed amount of biotinylated TirM_EHEC_ (2 pmols) in two-fold increasing volumes of PBS (from 0.1 to 1.5 ml) to reach a final concentration range from 20 nM to 1 nM. After this incubation, cells were washed and labeled with Streptavidin-PE as previously described. The relative MFI of the cells was plotted against the antigen concentration used and the curve fitted by non-linear least squares regression, giving an estimated apparent K_D_ of 1.7 x10^-9^ M (Figure 7C). This value is consistent with the estimated apparent K_D_ determined by SPR analysis, indicating that these *E. coli* display systems could also be used to estimate the K_D_ of selected sdAbs before purification.

## Discussion

In this work, we have demonstrated that the β-domains of EhaA and Intimin from EHEC O157:H7 [51] are effective platforms for the display sdAb libraries on the surface of *E. coli* K-12 cells, and allow the selection of high affinity sdAbs from immune libraries using biotinylated antigen and MACS. Despite their opposite topologies, both systems express stable fusion proteins with the native β-barrel correctly folded in the OM and display a functional sdAb with antigen-binding capacity on the surface of *E. coli* cells. Most sdAbs in the immune library were displayed on *E. coli* at good expression levels with both C-EhaA and Neae, with an average between 6000-8000 molecules/bacterium. From the immune libraries constructed in our study, we obtained a total of five independent camelid V_HH_ sequences binding the antigen used in the immunization (TirM_EHEC_), being the sdAb of higher affinity and specificity (VTIR1) the more frequent clone found in the selections with both β-domains. These results demonstrate that both *E. coli* display systems can be used to retrieve high-affinity binders from immune libraries of camelid V_HH_ sequences. Fewer different binders were found compared to other reported studies using an immune library of camelid V_HH_ displayed on the surface of phages and *Staphyloccous* *carnosus* cells [12]. However, we think that the relatively small number of binders obtained in our study reflects a limitation of the anti-TirM_EHEC_ library employed, and not of the *E. coli*-display systems described. Firstly, we have performed selections of the anti-TirM_EHEC_ V_HH_ library on phage that failed to retrieve other specific binders than those selected by *E. coli*-display (data not shown). Secondly, ongoing work in our laboratory with other immune V_HH_ libraries also seem to indicate that essentially the same pool of binders can be isolated from phage and *E. coli*-display systems. Thus, it appears that rather than the display system used, the actual diversity of binders retrieved from an immune V_HH_ library is more dependent on factors such as immunogenicity of the antigen, number of animals used in the immunization, and library size.

The small molecular weight, high protein solubility and stability of camelid V_HH_ domains are likely to help their effective translocation across the OM fused to the β-domains of EhaA and Intimin. Similar properties are also found in other natural sdAbs, like VNARs from sharks [52], and have been engineered in synthetic libraries based on human V_H_s and V_L_s [53-55]. Thus, most types of sdAbs could be efficiently displayed on *E. coli* cells with EhaA and Intimin β-domains. Larger Ab fragments based on a single polypeptide, such as scFvs, also have the potential to be displayed with these β-domains. However, the tendency of some scFv clones to oligomerize and aggregate may hinder their translocation across the OM [32]. Nonetheless, this could be advantageous for the selection of highly stable and soluble scFvs from scFv libraries. Lastly, Ab molecules with separate H and L polypeptide chains (e.g. Fabs, IgGs) cannot be displayed with the β-domains reported in this work (at least in their current configuration), and phage display and APEx should be used for their selection in *E. coli* [8,9,56].

Despite the functionality of the β-domains of EhaA and Intimin, we observed some important differences between these *E. coli* display systems. Firstly, NVHH fusions were found to be more stable than VHHA fusions *in vivo* (to *E. coli* proteases) and *in vitro* (to externally added proteases). This difference could be due to the natural resistance of Intimin to proteolysis and denaturation [25] and/or to the susceptibility of certain ATs to bacterial proteases as part of their secretion mechanism [24,26]. Secondly, expression of VHHA clones appeared to be more variable than NVHH fusions, with some clones showing significantly lower expression levels (e.g. VgfpA). This suggests that the N-terminal fragment of Neae could have a positive effect on the expression of sdAbs, similar to other N-terminal fusion partners (i.e. MBP, Trx1, GST) used for production of recombinant proteins and Ab fragments [57]. Thirdly, we found that the antigen-binding activity of NVHH fusions was at least 3-fold higher than that of VHHA fusions, as indicated by the flow cytometry signals of *E. coli* cells displaying these fusions when incubated with their cognate antigens (GFP or TirM_EHEC_). The lower antigen binding signals of *E. coli* cells displaying VHHA fusions was not explained by a different expression level and indicated the existence of additional factors. Although partial misfolding of VHHA fusions cannot be excluded, this possibility seems unlikely because both EhaA and Intimin β-domains use a common secretion pathway exposing the sdAb to periplasmic chaperones and DsbA before their translocation across the OM [25,33]. Alternatively, the longer linker region in NVHH fusions (with the D0 domain) could make the sdAb more accessible for the extracellular antigen by increasing its distance from the OM. The improved stability, expression and antigen-binding activity of NVHH fusions could explain why selection of TirM_EHEC_ binders was more efficient in the NVHH library than in the VHHA library, reaching a higher percentage of positive antigen binding clones in fewer selection rounds. From our data, the only limitation of the Neae display could be the ~40% reduction in viability of *E. coli* cultures expressing NVHH fusions (estimated as CFU/OD_600_). This reduction in viability does not have a significant effect on the representation of immune libraries with diversity ~10^7^ clones, since an excess of input bacteria over the Ab library size is used during MACS.

We chose MACS to select and recover *E. coli* cells bound to the antigen since this technology does not require the use of expensive cell-sorting equipment as in the case of FACS, and multiple samples along with controls can be processed in parallel. We employed a manual MACS system that can hold up to eight mini-MACS columns simultaneously, each with a capacity for ~10^8^ bacteria. In addition, MACS can be scaled up using multiple columns of higher capacity (each with a capacity of 10^9^-10^10^ bacteria) and it can be automated, which would allow the screening of large Ab libraries faster and more efficiently than FACS. Given the maximum density of *E. coli* cells that can be manipulated at ease (~2-3x10^10^ CFU/ml), naive and synthetic *E. coli* display libraries should have a maximum diversity between 10^9^-10^10^ clones in order to ensure a representation of all clones during selection. In fact, these numbers are very similar to the size of most naive and synthetic libraries of Ab fragments constructed in phage display vectors. Although bacteriophages can be produced and handled in higher densities than *E. coli* bacteria (i.e. ~10^13^ phages/ml) the diversity of phage display libraries is strongly limited by *E. coli* factors (i.e. transformation efficiency and the ability to infect cultures with sufficient number of bacteria). Hence, *E. coli* display is suitable for immune libraries with ~10^6^-10^7^ clones, as shown in this work, but it could also be applied to naive and synthetic libraries of ~10^9^ clones [54,55,58]. For screening of Ab libraries with higher diversities, phage display and, especially, cell-free display systems (e.g. *ribosome display*) would be more appropriate [4,59].

In conclusion, the *E. coli* cell display systems reported in this work represent a good alternative to phage display and APEx display systems for selection of positive binders from sdAb libraries. The major benefit of *E. coli* display over phage display is the use of flow cytometry for the direct determination of the expression levels and antigen binding specificity and affinity (K_D_) of the selected sdAb clones. Secondly, *E. coli* display makes possible the use of FACS, alone or in combination with MACS, for selections with antigen in solution. In addition, the multivalent Ab display and the less sticky properties of *E. coli* cells compared to filamentous bacteriophages, may reduce the background binding when complex antigenic surfaces (e.g. mammalian cells, tissues and organs) are used for selections. Although *E. coli* cells are more sensitive than bacteriophages to extremes of pH, temperature and other strong denaturants, *E. coli* cells having the OM can be washed with most common buffers and tolerate significant concentrations of detergents (e.g. 0.1-0.4% w/v) such as TX-100, SDS or deoxycholate [60]. This represents an improvement over the washing conditions tolerated by spheroplasts in APEx. Future work would aim to exploit the above-mentioned advantages of *E. coli* display for selections of novel Ab fragments of biomedical and biotechnological interest.

## Supporting Information

Figure S1 **Growth of *E. coli* cultures expressing VgfpA and NVgfp fusions.** (**A**) Growth curve of LB cultures of *E. coli* UT5600 cells carrying plasmids pVgfpA, pHEA (expressing C-EhaA), or pAK-Not (empty vector). (**B**) Growth curve of LB cultures of *E. coli* UT5600 cells carrying plasmids pNVgfp, pNeae2 (expressing Neae), or pAK-Not (empty vector). The cultures were incubated at 30 °C with agitation (160 rpm) and induced with 0.05 mM IPTG at the time indicated by an arrow. The optical density at 600 nm (OD_600_) of the cultures was monitored at the time points shown.
(TIF)

Figure S2 **Sensitivity of NVgfp fusion to Trypsin digestion.** Western blots of whole-cell protein extracts from IPTG-induced *E. coli* UT5600 cells harbouring pNVgfp. Intact *E. coli* cells were incubated with (+) or without (-) Trypsin before lysis. Protein extracts were prepared in SDS-urea sample buffer and boiled (+) or not (-) before loading onto SDS-polyacrylamide gels. Western blots were developed with anti-E or anti-myc mAb, as indicated. The positions of full-length NVgfp are labeled with arrows. The protein band with faster mobility corresponds to the folded conformation of the polypeptide. Mass of protein standards is shown on the left (in kDa).
(TIF)

Figure S3 **Quantification of the number of VHHA and NVHH fusions expressed in *E. coli*.** The plot shows the intensity of protein bands from Western blots developed with anti-E-tag mAb and quantified on a ChemiDoc XRS using the Quantity One software (Bio-Rad). Samples analyzed were whole-cell protein extracts from ~1.5x10^8^ bacteria (0.15 units of OD_600_) of induced *E. coli* EcM1 cells carrying the pVHHA or pNVHH anti-TirM_EHEC_ libraries. The standard curve was generated with the values of band intensities (Intensity/mm^2^) of a purified E-tagged V_HH_ of known concentration. Protein samples and protein standards were loaded in duplicates and the average values of band intensities were plotted. Two independent experiments were done with similar results.
(TIF)

Figure S4 **Growth of *E. coli* cultures expressing VHHA and NVHH anti-TirM_EHEC_ libraries.** Growth curve of LB cultures of *E. coli* EcM1 cells carrying pAK-Not (empty vector) or plasmids of the pVHHA and pNVHH anti-TirM_EHEC_ libraries. The cultures were incubated at 30 °C with agitation (160 rpm) and induced with 0.05 mM IPTG at the time indicated by an arrow. The optical density at 600 nm (OD_600_) of the cultures was monitored at the time points shown.
(TIF)

Figure S5 ***E. coli* cell surface display levels of VTIR1A and NVTIR1 clones.** Fluorescent flow cytometry analysis of induced *E. coli* EcM1 cells expressing VTIR1A or NVTIR1 clone (as indicated). Control cells carried the empty vector pAK-Not. Histograms show the fluorescence intensity of bacteria stained with anti-E mAb and secondary anti-mouse IgG-Alexa 488.
(TIF)

Figure S6 **Monomeric behaviour and binding activity of the purified sdAb VTIR1.** (**A**) Gel-filtration chromatograms of sdAbs VTIR1 and Vgfp purified from the periplasm of *E. coli* WK6 cells (carrying the corresponding pCANTAB6-derivative) after a metal-affinity chromatography step. Gel-filtration chromatography was performed in a HiLoad 16/600 Superdex 75 column calibrated with protein markers (labeled in kDa) and Blue dextran (for exclusion volume Vo). Both sdAbs have major peaks of ~15 kDa corresponding to their monomeric forms. (**B**) ELISA of purified monomeric VTIR1 and Vgfp (control) against TirM_EHEC_ and BSA. The plot represents the OD values at 490 nm obtained with the indicated concentrations of sdAbs. ELISA developed with anti-myc mAb-POD as secondary.
(TIF)

Materials and Methods S1 (DOCX)
