# Supplementary figures and images for: Selection of Single Domain Antibodies from Immune Libraries Displayed on the Surface of E. coli Cells with Two β-Domains of Opposite Topologies
Source: PLoS One. 2013 Sep 23;8(9):e75126. doi: 10.1371/journal.pone.0075126 (PMC3781032; doi:10.1371/journal.pone.0075126)

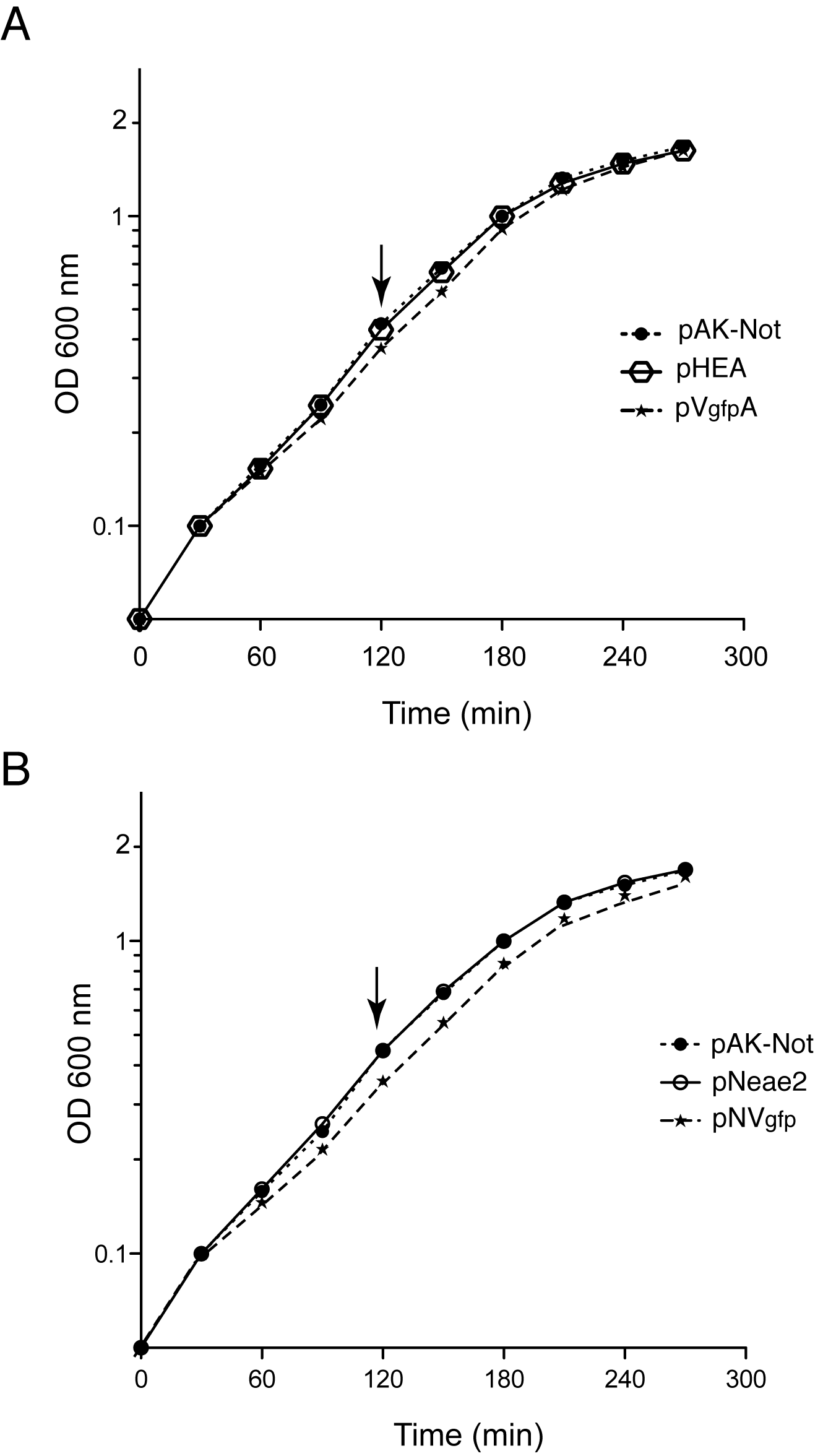

Supplement: Figure S1 — Growth of E. coli cultures expressing VgfpA and NVgfp fusions. (A) Growth curve of LB cultures of E. coli UT5600 cells carrying plasmids pVgfpA, pHEA (expressing C-EhaA), or pAK-Not (empty vector). (B) Growth curve of LB cultures of E. coli UT5600 cells carrying plasmids pNVgfp, pNeae2 (expressing Neae), or pAK-Not (empty vector). The cultures were incubated at 30 °C with agitation (160 rpm) and induced with 0.05 mM IPTG at the time indicated by an arrow. The optical density at 600 nm (OD600) of the cultures was monitored at the time points shown. (TIF) [file pone.0075126.s001.tif]

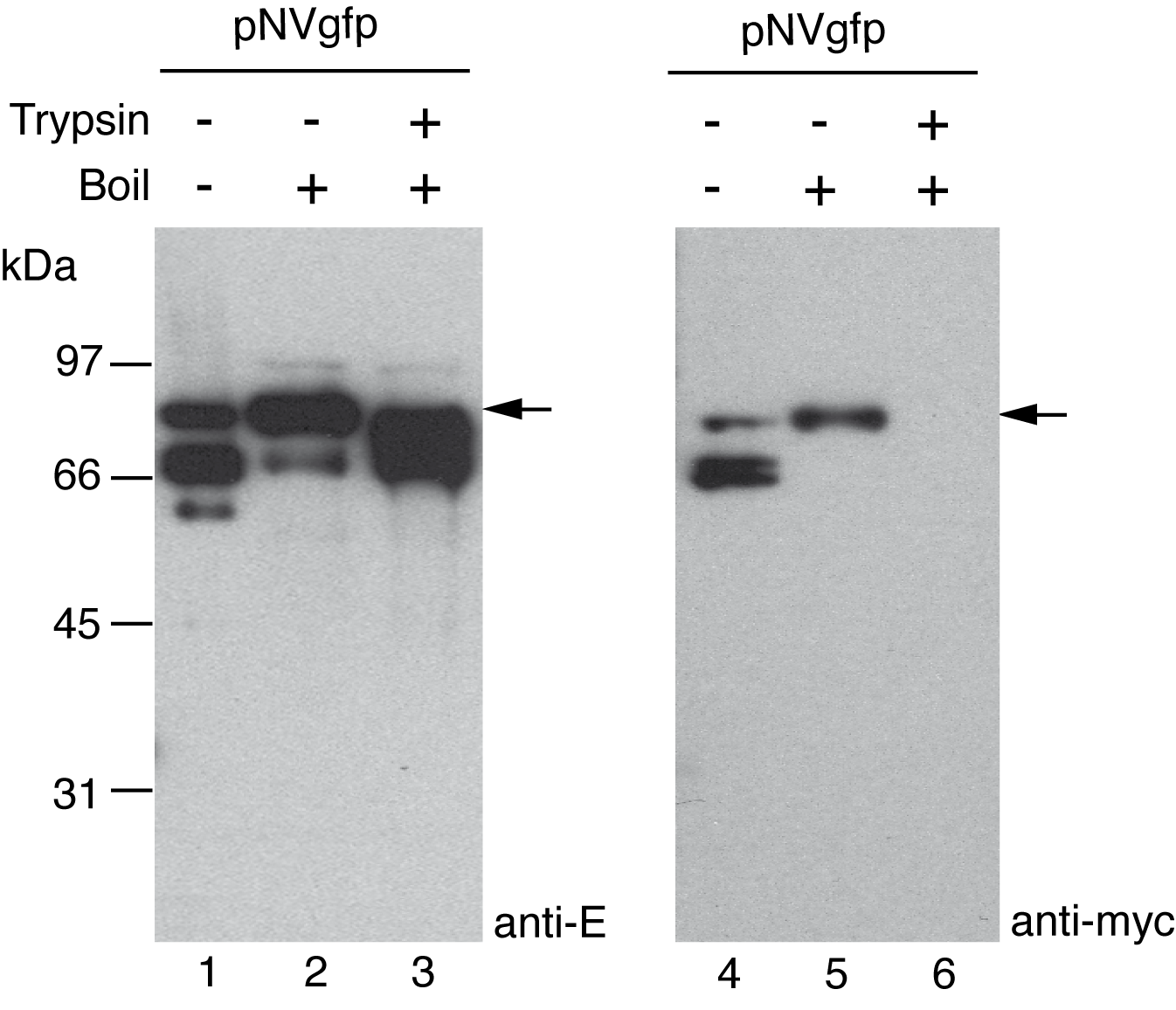

Supplement: Figure S2 — Sensitivity of NVgfp fusion to Trypsin digestion. Western blots of whole-cell protein extracts from IPTG-induced E. coli UT5600 cells harbouring pNVgfp. Intact E. coli cells were incubated with (+) or without (-) Trypsin before lysis. Protein extracts were prepared in SDS-urea sample buffer and boiled (+) or not (-) before loading onto SDS-polyacrylamide gels. Western blots were developed with anti-E or anti-myc mAb, as indicated. The positions of full-length NVgfp are labeled with arrows. The protein band with faster mobility corresponds to the folded conformation of the polypeptide. Mass of protein standards is shown on the left (in kDa). (TIF) [file pone.0075126.s002.tif]

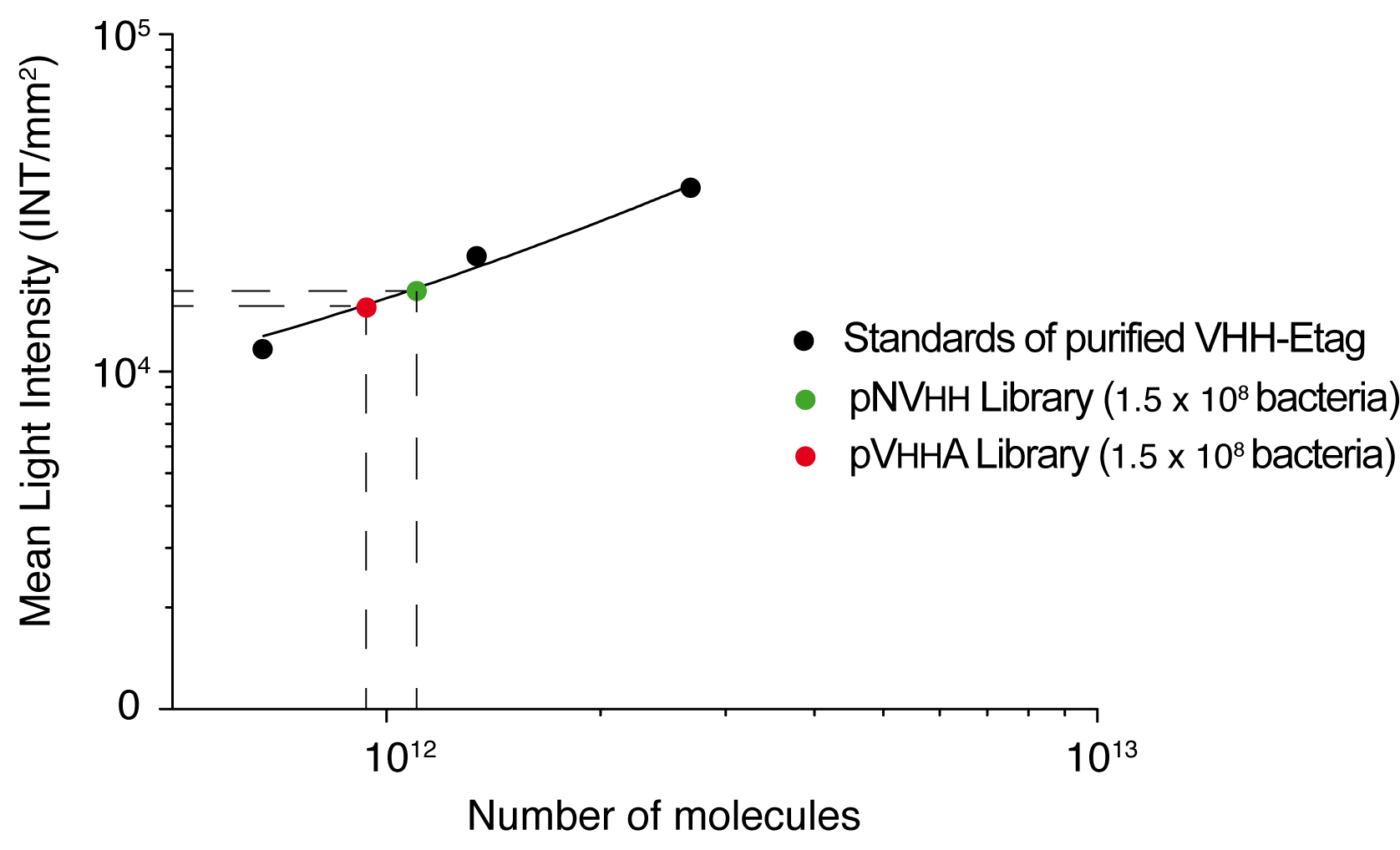

Supplement: Figure S3 — Quantification of the number of VHHA and NVHH fusions expressed in E. coli. The plot shows the intensity of protein bands from Western blots developed with anti-E-tag mAb and quantified on a ChemiDoc XRS using the Quantity One software (Bio-Rad). Samples analyzed were whole-cell protein extracts from ~1.5x10^8^ bacteria (0.15 units of OD600) of induced E. coli EcM1 cells carrying the pVHHA or pNVHH anti-TirMEHEC libraries. The standard curve was generated with the values of band intensities (Intensity/mm2) of a purified E-tagged VHH of known concentration. Protein samples and protein standards were loaded in duplicates and the average values of band intensities were plotted. Two independent experiments were done with similar results. (TIF) [file pone.0075126.s003.tif]

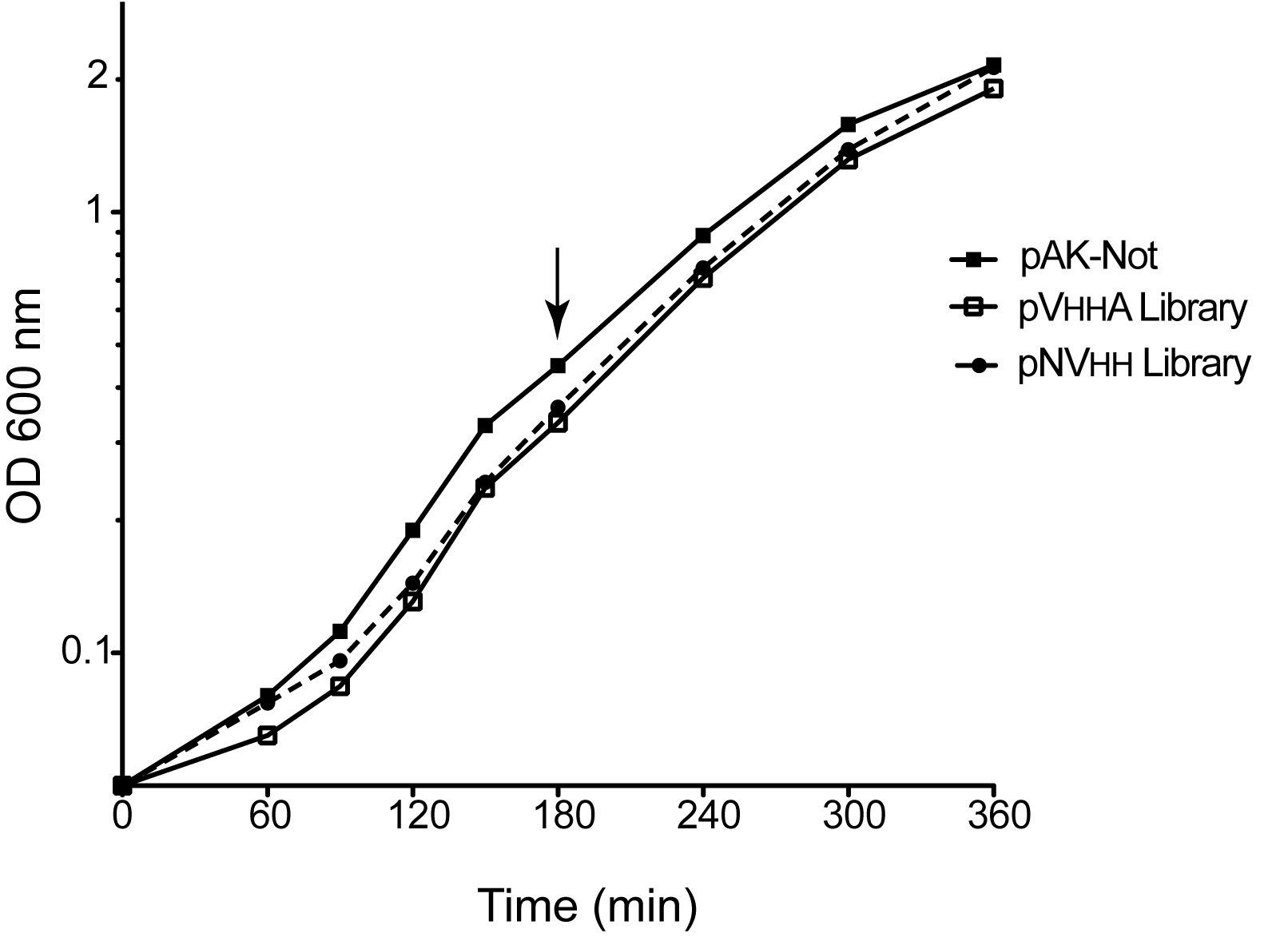

Supplement: Figure S4 — Growth of E. coli cultures expressing VHHA and NVHH anti-TirMEHEC libraries. Growth curve of LB cultures of E. coli EcM1 cells carrying pAK-Not (empty vector) or plasmids of the pVHHA and pNVHH anti-TirMEHEC libraries. The cultures were incubated at 30 °C with agitation (160 rpm) and induced with 0.05 mM IPTG at the time indicated by an arrow. The optical density at 600 nm (OD600) of the cultures was monitored at the time points shown. (TIF) [file pone.0075126.s004.tif]

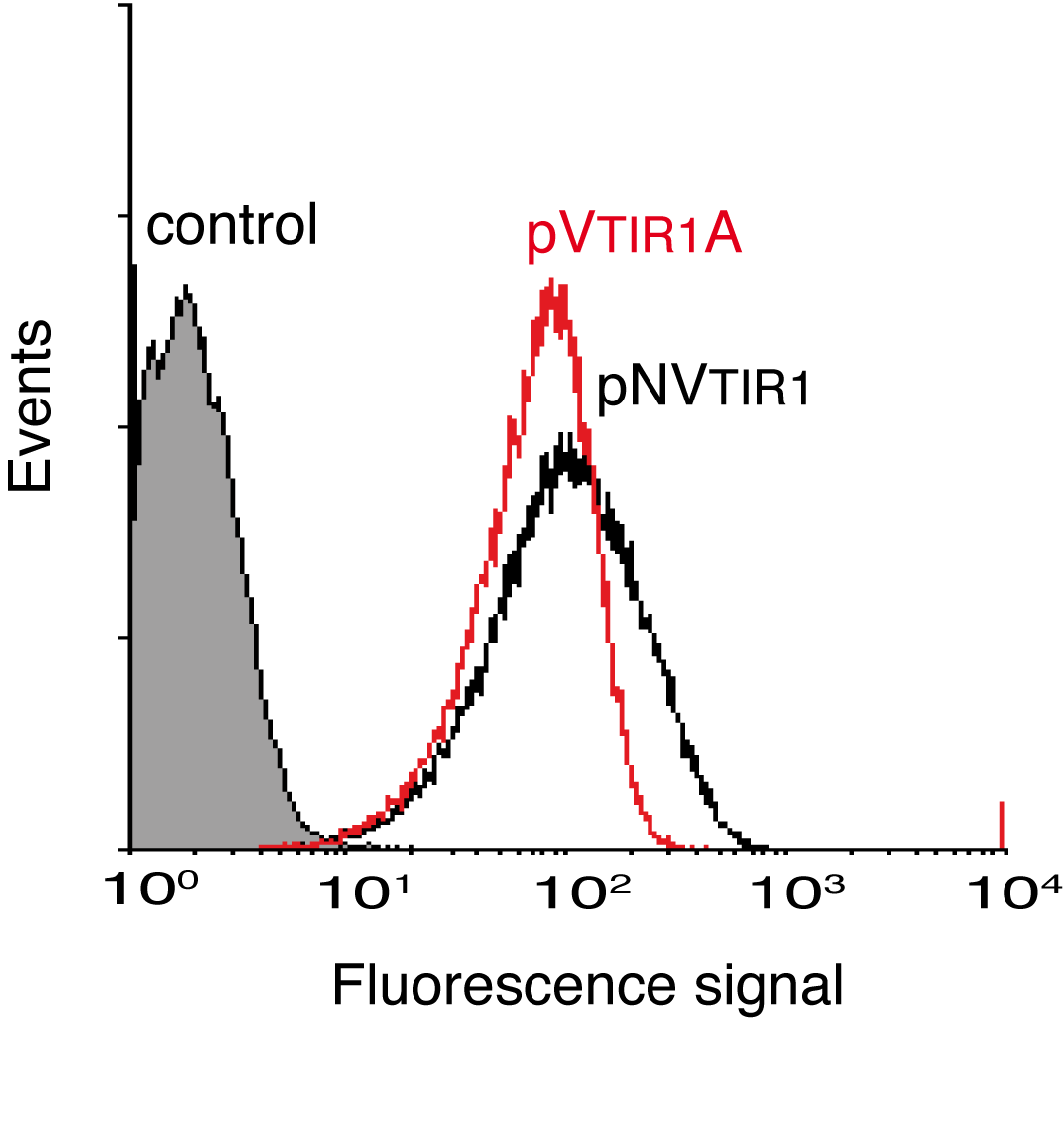

Supplement: Figure S5 — E. coli cell surface display levels of VTIR1A and NVTIR1 clones. Fluorescent flow cytometry analysis of induced E. coli EcM1 cells expressing VTIR1A or NVTIR1 clone (as indicated). Control cells carried the empty vector pAK-Not. Histograms show the fluorescence intensity of bacteria stained with anti-E mAb and secondary anti-mouse IgG-Alexa 488. (TIF) [file pone.0075126.s005.tif]

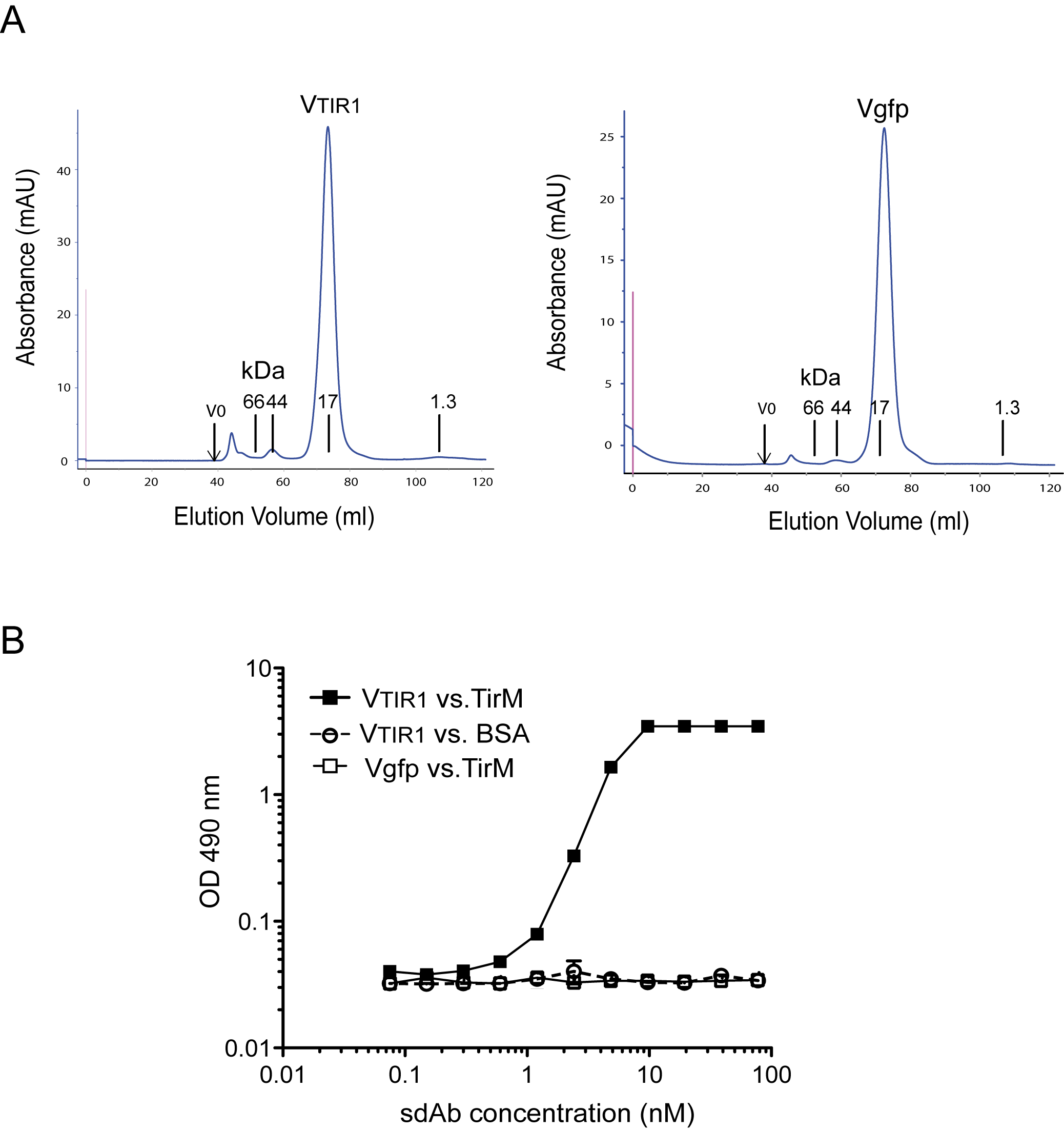

Supplement: Figure S6 — Monomeric behaviour and binding activity of the purified sdAb VTIR1. (A) Gel-filtration chromatograms of sdAbs VTIR1 and Vgfp purified from the periplasm of E. coli WK6 cells (carrying the corresponding pCANTAB6-derivative) after a metal-affinity chromatography step. Gel-filtration chromatography was performed in a HiLoad 16/600 Superdex 75 column calibrated with protein markers (labeled in kDa) and Blue dextran (for exclusion volume Vo). Both sdAbs have major peaks of ~15 kDa corresponding to their monomeric forms. (B) ELISA of purified monomeric VTIR1 and Vgfp (control) against TirMEHEC and BSA. The plot represents the OD values at 490 nm obtained with the indicated concentrations of sdAbs. ELISA developed with anti-myc mAb-POD as secondary. (TIF) [file pone.0075126.s006.tif]
