# Supplementary material for: Selection of Single Domain Antibodies from Immune Libraries Displayed on the Surface of E. coli Cells with Two β-Domains of Opposite Topologies
Source: PLoS One. 2013 Sep 23;8(9):e75126. doi: 10.1371/journal.pone.0075126 (PMC3781032; doi:10.1371/journal.pone.0075126)
Supplement: Materials and Methods S1 — (DOCX) [file pone.0075126.s007.docx]

**Supporting Information**

**Selection of single domain antibodies from immune libraries displayed on the surface of *E. coli* cells with two β-domains of opposite topologies**

Valencio Salema^#1^, Elvira Marín^#1^, Rocio Martínez-Arteaga^1^, David Ruano-Gallego^1^,

Sofía Fraile^1^, Yago Margolles^1^, Xema Teira^1^, Carlos Gutierrez^2^, Gustavo Bodelón^1^,

and Luis Ángel Fernández^1^*

*(1) Department of Microbial Biotechnology, Centro Nacional de Biotecnología, Consejo Superior de Investigaciones Científicas (CSIC), Campus UAM Cantoblanco, Madrid, Spain.*

*(2) Department of Animal Medicine and Surgery, Veterinary Faculty, Universidad de Las Palmas de Gran Canaria (UPGC), Las Palmas, Canary Islands, Spain.*

***Keywords*:** Antibody libraries/ Autotransporters/ Intimin/ surface display/ *E. coli* /single domain antibodies/

Note: # These authors contribute equally to this work

___________________________________________________________________

*Corresponding Author: Dr. Luis Ángel Fernández

Centro Nacional de Biotecnología, CNB- CSIC

Darwin 3

Campus UAM, Cantoblanco

Madrid 28049 (Spain).

Phone: +34 91 585 48 54

Fax: +34 91 585 45 06

E-mail: [lafdez@cnb.csic.es](mailto:lafdez@cnb.uam.es)

**Supporting Materials and Methods**

**Growth and induction conditions**. Preinoculum of *E. coli* cells (UT5600 or EcM1 strains, as indicated) with pVHHA and pNVHH plasmids (Cm^R^) were grown in 5 ml LB medium containing 2% (w/v) glucose and Cm overnight (o/n). The next day, the preinocula were centrifuged, bacteria washed with LB to remove glucose, and diluted to a final OD_600_ of 0.5 in 10 ml of fresh LB medium with Cm and containing 0.05 mM of isopropylthio-β-D-galactoside (IPTG) for induction, and grown for 3 h at 30 ^o^C with agitation (160 rpm). To follow the growth curve, washed bacteria from the pre-inoculum were diluted to a final OD_600_ of 0.05 in 10 ml of fresh LB medium with Cm, incubated at 30 ^o^C with agitation until OD_600_ was 0.5, and then induced with IPTG under the same conditions.

**DNA constructs and oligonucleotides.** Plasmid pNeae2 (Cm^R^) was constructed by amplificaiton of DNA encoding the 3' region of Neae with C-terminal myc-tag by PCR with oligonucleotides eae4 5’-CGT AAT GGC AAT AGC TCT AAC AAT GTA-3’ and His-Not-myc-stop-H3 (5’-GCA GGA AGC TTT TAT GCA GCT GCA TCC TCT TCT GAG ATG AGT TTT TGT TCG GCG GCC GCG TGA TGG TGA TGG TG-3’) using original pNeae as DNA template [[1](#_ENREF_1)]. The amplified fragment (~0.5 kb) was digested with *Sac*II and *Hind*III, and ligated into the same sites of pNeae vector backbone, replacing the original 3' region of Neae. Plasmids pVgfpA (Cm^R^), and pNVgfp (Cm^R^), were constructed by ligation of the DNA fragment encoding the V_HH_ anti-GFP, obtained by digestion of pT3sVgfp [[2](#_ENREF_2)] with *Sfi*I and *Not*I (~0.4 kb), and ligated into the same sites of pHEA and pNeae2 vector backbones, replacing the His epitope encoded in both vectors between the *Sfi*I and *Not*I sites [[1](#_ENREF_1),[3](#_ENREF_3)]. For secretion into *E. coli* culture media, VHH genes were cloned as *Sfi*I-*Not*I fragments in the same sites of vector pEHlyA4SD, which is derived from pEHlyA2SD [[4](#_ENREF_4)] with a modified polylinker containing unique *Sfi*I and *Not*I sites upstream the E-tagged C-terminal of HlyA. The polylinker sequence was inserted between *Nco*I-*Xma*I sites of pEHlyA2SD [[4](#_ENREF_4)] resulting in the following sequence: 5'-CCATGGTGGCCCAGCCGGCCACTAGTGAGCTCGCGGCCGCATCGGGGGCCGCGTCGACGCACCATCACCATCACCATGCTTCGACGCCCGGG-3'. Plasmids pCANTAB6-Vgfp (Ap^R^) and pCANTAB6-VTIR1 (Ap^R^), were constructed by ligation of the ~0.4 kb DNA fragment encoding the V_HH_ anti-GFP and anti-TirM_EHEC_, digested *Sfi*I and *Not*I from pNVgfp and pNVTIR1, and cloned into the same sites of pCANTAB6 [[5](#_ENREF_5)]. Plasmid pET28a-TirM_EHEC_ (Km^R^), for overexpression with T7 promoter of soluble TirM_EHEC_ with N-terminal His-tag, was constructed by amplification of a ~0.35 kb DNA fragment encoding residues 252 to 360 of full-length Tir_EHEC_ from chromosomal DNA from EHEC O157:H7 strain EDL933*stx*- [[6](#_ENREF_6),[7](#_ENREF_7)] with oligonucleotides BamEcoTirM-EHEC (5'-CGC GGA TCC GAA TTC CAG GCG CTT GCA TTG ACG CCG GAG-3') XhoHindTirM-EHEC (5'- CCG CTC GAG AAG CTT TTA CGA TGA AAC TTT CAG CTC CTC CTG-3'), and cloned as *Eco*RI -*Hind*III fragment in pET28a (Km^R^; Novagen).

**Immunization and generation of VHH sdAb libraries.** Purified TirM_EHEC_ (~1 mgr) was diluted in sterile water to 10 ml final volume and stored in 2 ml aliquots at -20 ^o^C until its use. An aliquot of antigen was mixed with the same volume of adjuvant (Veterinary Vaccine Adjuvant, GERBU) and injected subcutaneously to one 14-years-old male dromedary camel (*Camelus dromedarius*) each week during 5 weeks. Pre-immune serum was prepared from a small blood sample (5 ml) before the first injection. Seven days after the last injection, immune serum was prepared similarly and additional 50 ml of blood were collected from jugular vein in tubes containing EDTA as anticoagulant using Venoject system. Serial dilutions of pre-immune and immune sera were used in ELISA to confirm Ab immune response against TirM_EHEC_ with protein-A-POD as secondary. For lymphocyte isolation, the anti-coagulated blood sample was mixed with the same volume of RPMI-1640 medium (Sigma), divided in 4 aliquots of 25 ml and each aliquot added on top of a 25 ml of Ficoll-Paque Plus (StemCell Technologies) in 50 ml sterile Falcon tubes (BD Biosciences). After centrifugation (800 xg, 30 min, RT), lymphocytes was taken from the interphase, washed twice in RPMI-1640 by centrifugation (800 xg, 10 min), resuspended in 5 ml of RPMI-1640, and the number of cells determined in a Neubauer hematocytometer (Hausser Scientific). About 2x10^7^ cells were lysed with 2 ml of TRIzol (Invitrogen) for RNA extraction following manufacturer instructions. The poly-A^+^ mRNA was purified from total RNA using an oligo-dT resin (Oligotex mRNA Minikit, Qiagen) and directly employed as template for first-strand cDNA synthesis reactions with random hexamers and oligo-dT primers (iScript cDNA Synthesis, Bio-Rad). Typically, ~1 μl of each cDNA synthesis was used as template in 50 μl PCR reactions with oligonucleotides CALL001 (5’-GTC CTG GCT CTC TTC TAC AAG G-3’) and CALL002 (5’-GGTACGTGCTGTTGAACTGTTCC-3’). The amplified fragments of ~0.6 kb, corresponding to VHH-CH2 domains, and ~0.9 kb, corresponding to conventional VH-CH1-CH2 domains, were separated in 1.2% (w/v) low melting agarose gel and the ~0.6 kb band was purified (Qiaex II Gel Extraction kit, Qiagen). This fragment was used as template in a second PCR reaction with oligonucleotides Vhh-Sfi2 (5’-GTC CTC GCA ACT GCG GCC CAG CCG GCC ATG GCT CAG GTG CAG CTG GTG GA-3’) and Vhh-Not2 (5’-GGA CTA GTG CGG CCG CTG AGG AGA CGG TGA CCT GGG T-3’) to finally obtain the amplified fragments of ~0.4 kb, corresponding to VHH domains. The amplified VHH fragments were digested with *Sfi*I and *Not*I restriction enzymes and first ligated into the same sites the phagemid pCANTAB5E backbone [[5](#_ENREF_5)]. The intermediate cloning step in the phagemid was included to ensure that both *E. coli* display vectors receive a similar pool of VHH fragments. Ligations were electroporated in *E. coli* TG1 cells (Stratagene) and a library size of ~2x10^6^ clones was determined by plating on LB-Ap agar plates with 2% w/v glucose incubated at 30 ^o^C. Transformed bacteria were recovered in 5 ml of LB liquid media, harvested by centrifugation, and ~50 units of OD_600_ were used for plasmid isolation (NucleoBond Xtra Midi, Macherey-Nagel). The VHH fragments were isolated after digestion with *Sfi*I and *Not*I restriction enzymes and ligated into the same sites of purified pHEA and pNeae2 backbone vectors. Ligations were electroporated in *E. coli* DH10B-T1^R^ cells. The size of each library was ~2-3x10^6^ clones, as determined by plating on LB-Cm agar plates with 2% w/v glucose incubated at 30 ^o^C. Fewer than 5% re-ligated vectors were estimated from parallel control ligations without DNA insert and DNA sequencing of 40 clones picked randomly from each library. Transformed bacteria were scraped from plates in LB, their plasmids isolated and electroporated into *E. coli* EcM1 cells. About 1x10^7^ colonies from each library were harvested from plates, grown and induced with IPTG.

**Protein extracts, SDS-PAGE and Western blots.** Whole cell protein extracts were prepared by harvesting bacteria after induction (1 ml of OD_600_ 1.5), resuspended in 50 μl of 10 mM Tris HCl pH 8.0, mixed with the same volume of SDS-sample buffer (2X) or urea-SDS sample buffer (2X) and boiled for 10 min (pHEA constructs) or 30 min (pNeae constructs). The SDS-sample buffer (1X) consists of 60 μM Tris-HCl pH 6.8, 1% w/v SDS, 5% (v/v) glycerol, 0.005% (w/v) bromophenol blue and 1% (v/v) 2-mercaptoethanol (2-ME). The urea-SDS-sample buffer (1X) contains 60 μM Tris-HCl pH 6.8, 2% w/v SDS, 4 M urea, 5 mM EDTA, 5% (v/v) glycerol, 0.005% (w/v) bromophenol blue and 1% (v/v) 2-mercaptoethanol (2-ME). The boiled samples were sonicated (5 sec; Labsonic B Braun), centrifuged (14,000 x*g*, 5 min) to pellet insoluble material, loaded onto 8% or 10% SDS-PAGE gels and run using a Miniprotean III electrophoresis system (Bio-Rad). For Western blot, the gels were transferred to a polyvinylidene difluoride membrane (PVDF, Immobilon-P, Millipore) using a semi-dry electrophoresis transfer apparatus (Bio-Rad) and the membranes were blocked in phosphate buffered saline (PBS; 8 mM Na_2_HPO_4_, 1.5 mM KH_2_PO_4_, 3 mM KCl, 137 mM NaCl, pH 7.0) with 3% (w/v) skimmed milk (Milk-PBS) for 1 hour at RT. For immunodetection of the E-tagged proteins, membranes were incubated for 1 h at room temperature (RT) in the same buffer with anti-E-tag mAb (Phadia), while for immunodetection of the myc-tagged proteins anti-c-myc-POD mAb (clone 9E10; Roche) was used. Membranes were washed three times with PBS containing 0.1% Tween 20 to remove unbound antibodies. Bound anti-E-tag mAb was developed using anti-mouse IgG conjugated with peroxidase (POD) (Sigma). Streptavidin-POD conjugate (Roche) was employed to detect the biotinylated broad range SDS-PAGE protein markers (Bio-Rad). All mAbs and POD conjugates were used in a 1:5000 dilution. For developing, a chemiluminescence reaction was prepared using a mixture of 1.25 mM luminol (Sigma) and 200 μM p-coumaric acid (Sigma) in 10 ml of 100 mM Tris–HCl (pH 8.0). Following a rapid rinse in PBS, the membranes were soaked in the chemiluminiscence mixture and H_2_O_2_ (Sigma) was added at 0.02% (v/v), followed by one minute incubation in the dark and the PVDF-membranes were either exposed to an X-ray film (Curix, Agfa) or scanned in a Chemi-Doc XRS (Bio-Rad). To quantify the total number of VHH fusions expressed in *E. coli*, Western blots of whole cell protein extracts and dilutions of a purified E-tagged V_HH_ of known concentration [[2](#_ENREF_2)] (hereafter referred to as “unknowns” and “standard”, respectively) were visualized on a ChemiDoc XRS and analyzed using the Quantity One software (Bio-Rad). The intensity of bands from standard and unknown samples was measured and the local background was corrected. The total number of molecules in the various dilutions of the standard protein was calculated and was plotted against the corresponding values of density of its band (Intensity/mm^2^) to generate a standard curve. Based upon the standard curve plot, the number of molecules of VHH fusions in the unknown samples was estimated, assuming that ~1.5x10^8^ bacteria (0.15 units of OD_600_) were loaded per lane. Two independent experiments were done with each sample in duplicates.

**Purification of TirM_EHEC_*.*** Induced *E. coli* BL21(DE3) cells carrying pET28a-TirM_EHEC_ from 500 ml culture were centrifuged (4000 xg, 15 min, 4ºC) and the pellet was resuspended in 25 ml of PN3 buffer (50 mM sodium phosphate pH 7.4, 300 mM NaCl) containing DNase (0.1 mg/ml; Roche) and a cocktail of protease inhibitors (Complete EDTA-free; Roche). The cells were lysed in a French Press at 1200 psi (3 cycles) and the whole cell lysate was ultracentrifuged (40000 xg, 1 h, 4ºC). The supernatant was loaded (flow rate of 1 ml/min) onto a column packed with 3 ml of a Cobalt-containing resin (Talon, Clontech) pre-equilibrated with 30 ml PN3 buffer. The column was subsequently washed with 10 ml of PN3 buffer, followed by a wash with 10 ml of PN3 buffer containing 5 mM imidazole and the protein was finally eluted in 1 ml fractions with the PN3 buffer containing 150 mM imidazole. The eluted fractions were dialyzed against HEPES-buffer (20 mM HEPES pH 7.4, 200 mM NaCl, sterile filtered and degassed). Dialyzed fractions were concentrated 10 fold in a 3-kDa centrifugal filter unit (Amicon Ultra-15) and loaded onto a gel filtration column (HiLoad 16/600 Superdex 75 preparative grade, GE Healthcare), pre-equilibrated with HEPES-buffer and calibrated with protein markers (Gel Filtration Standards, Bio-Rad) and Blue dextran (for exclusion volume Vo; Sigma). Fractions of 1 ml containing TirM_EHEC_ were collected and checked for purity by SDS-PAGE. Protein concentration was estimated using the Bicinchoninic acid (BCA) Pierce protein assay kit (Thermo Scientific).

**References of Supporting Information**

1. Bodelón G, Marín E, Fernández LA (2009) Role of periplasmic chaperones and BamA (YaeT/Omp85) in folding and secretion of Intimin from enteropathogenic *Escherichia coli* Strains. J Bacteriol 191: 5169-5179.

2. Blanco-Toribio A, Muyldermans S, Frankel G, Fernández LA (2010) Direct injection of functional single-domain antibodies from *E. coli* into human cells. PLoS ONE 5: e15227.

3. Marín E, Bodelón G, Fernández LA (2010) Comparative analysis of the biochemical and functional properties of C-terminal domains of autotransporters. J Bacteriol 192: 5588-5602.

4. Fernández LA, Sola I, Enjuanes L, de Lorenzo V (2000) Specific secretion of active single-chain Fv antibodies into the supernantants of *Escherichia coli* cultures by use of the hemolysin system. Appl Environ Microbiol 66: 5024-5029.

5. McCafferty J, Johnson KS (1996) Construction and screening of antibody display libraries. In: Kay BK, Winter J, McCafferty J, editors. Phage display of peptides and proteins. San Diego, California: Academic Press, Inc. pp. 79-111.

6. Garmendia J, Phillips AD, Carlier MF, Chong Y, Schuller S, et al. (2004) TccP is an enterohaemorrhagic Escherichia coli O157:H7 type III effector protein that couples Tir to the actin-cytoskeleton. Cell Microbiol 6: 1167-1183.

7. Perna NT, Plunkett G, 3rd, Burland V, Mau B, Glasner JD, et al. (2001) Genome sequence of enterohaemorrhagic Escherichia coli O157:H7. Nature 409: 529-533.
